# Implementing the immunization agenda 2030: A framework for action through coordinated planning, monitoring & evaluation, ownership & accountability, and communications & advocacy^☆^

**DOI:** 10.1016/j.vaccine.2021.09.045

**Published:** 2024-04-08

**Authors:** Ann Lindstrand, Eric Mast, Sarah Churchill, Nargis Rahimi, Jan Grevendork, Alan Brooks, Edda Magnus, Robin Nandy, Katherine L. O'Brien

**Affiliations:** a Department of Immunization, Vaccines and Biologicals, World Health Organization, Geneva, Switzerland; b Global Immunization Division, Centers for Disease Control and Prevention, Atlanta, USA; c Bridges to Development, Geneva, Switzerland; d Department of Health Systems Governance and Financing, World Health Organization, Geneva, Switzerland; e Program Division, United Nations Children's Fund (UNICEF), NY, USA

**Keywords:** Vaccines, IA2030, Monitoring, Evaluation, Ownership, Accountability, Communications, And advocacy

## 1 Introduction

In November 2020, the Seventy-Third World Health Assembly endorsed the Immunization Agenda 2030: A Global Strategy to Leave No One Behind (IA2030) in decision WHA73/(9). IA2030 defines what needs to happen to achieve the global vision of *a world where everyone, everywhere, at every age fully benefits from vaccines for good health and well-being*.

IA2030 is a global strategy created for the global community and requiring broad ownership by all immunization and non-immunization stakeholders, including those involved in health system strengthening and disease-specific initiatives. While WHO was asked to lead the development of IA2030, all stakeholders co-created, co-developed and now co-own it. IA2030 has been designed to respond to the interests of each and every country, regardless of income level or geography. Recognizing that the most important actions for success must be taken by individual Member States, IA2030 aims to reinforce country ownership for planning and implementing effective and comprehensive vaccination programmes.

IA2030 will become operational through four critical elements:

• regional and national strategies (**operational planning**);
• a mechanism to ensure **ownership and accountability** (O&A);
• a **monitoring and evaluation** (M&E) framework to guide implementation;
• and **communication and advocacy** (C&A), to ensure that immunization remains high on the health agenda and to rally support for IA2030.

At this pivotal moment for immunization, implementation of IA2030 will initially focus on a comprehensive response to the COVID-19 pandemic and a repair to the damage it has caused. An urgent priority is the rapid and equitable scale-up of COVID-19 vaccines in all countries. For the many countries without adult immunization programmes, this presents a major challenge. In addition, the current focus on COVID-19 draws resources away from existing vaccination activities, requiring countries to address the disruption to their immunization and other essential primary health care services.

These challenges set the immediate priorities for IA2030 implementation. IA2030 will support urgent collective action to catch up on missed vaccinations and rebuild essential services. This will include intensification of routine services to catch up on children who missed vaccine doses through context appropriate strategies and the implementation of supplementary immunization activities or campaigns where necessary. IA2030’s commitment to eliminating equity gaps, particularly reducing the numbers of “zero-dose” children (those not receiving any essential vaccines), by focusing on communities where large numbers of zero dose children are clustered in, will be more important than ever as countries wrestle with the dual challenges of introducing COVID-19 vaccination and maintaining and strengthening existing immunization programmes. Children in the most deprived communities, such as remote rural settings, urban slums and conflict-affected communities must not be left behind as the world recovers from COVID-19.

Rebuilding of immunization programmes in this way will also make a major contribution to the strengthening of primary health care systems. Effective childhood and adult immunization programmes, including COVID-19, will lie at the heart of resilient and sustainable primary health care systems that will be central to future global health security.

### 1.1 Purpose

The purpose of this Framework for Action is to describe how each of the four critical elements will be integrated to ensure successful implementation of the IA2030 strategy to achieve the IA2030 vision.

The document first summarizes a set of overarching considerations, and then addresses the following aspects:

• How the four critical elements work as a Framework for Action (Section 2).
• How they will implementated at country, regional and global levels (Section 3).
• Considerations in the current context of COVID-19 (Section 4).
• How a Learning Agenda will help inform the path ahead (Section 5).

An Annex provides a more detailed description of the M&E component.^1^

First prepared in November 2020, this document has been updated to reflect feedback from consultations to date with WHO Member States^2^ and the WHO Executive Board, as well as input from other stakeholders.

As a living document, this guidance will be updated based on early implementation experience, new priorities and challenges, and likely needs during the next decade. In particular, IA2030 indicators will require critical review and adaptation in light of the evolving COVID-19 pandemic and its effect on immunization programmes. The IA2030 Learning Agenda provides an initial framework for updating this document.

### 1.2 IA2030 co-development

During 2019, the IA2030 strategy and vision core document was co-developed with Member States and partners committed to improving immunization outcomes. This co-development approach continued in 2020 and 2021, and has underpinned the development of the operational elements described in this paper.

Implementation planning for IA2030 draws on the lessons learned from the Global Vaccine Action Plan (GVAP)^3^. In addition, each of the four operational elements has been shaped by broad stakeholder inputs:

1. Development of the **Ownership & Accountability** model and **operational planning** **guidance** has been led by the core team of IA2030 partners^4^. Extensive consultations were held in July and August 2020 with a diverse range of stakeholders, including senior government officials, national immunization programme managers, and representatives from National Immunization Technical Advisory Groups (NITAGs), academia, non-health sectors, civil society organizations (CSOs), and development partners from low-, middle- and high-income countries.
2. The **Monitoring & Evaluation** approach has been developed by a task force with representatives from countries and regions, in collaboration with core IA2030 partners, the seven IA2030 strategic priority Working Groups, and in consultation with a “sounding board” that included additional representatives from countries, WHO Regional Offices, the WHO Strategic Advisory Group of Experts (SAGE), academia and CSOs. At its October 2020 meeting, SAGE reviewed draft O&A and M&E models. This document incorporates revisions recommended by SAGE, as well as additional input from development partners.
3. A **Communications and Advocacy** (C&A) strategy was co-created with input from immunization partners, communications and advocacy experts, and CSOs at the country, regional and global level. Input was gathered through national and regional surveys, interviews, focus group discussions and from the extensive O&A national consultations. The co-created strategy is now being operationalized through a collaborative effort to bring to life the proposed launch activities including a messaging framework and structures to ensure continuous engagement throughout the decade.
4. Each Region has developed their regionally adapted version of IA2030 through extensive consultations across all relevant stakeholders, and National Immunization Strategies are being developed in many countries focused on the context and priorities.

### 1.3 Guiding principles

The Framework for Action draws on the following principles:

• Instilling broad ownership to achieve the IA2030 vision among all immunization and non-immunization stakeholders, including those involved in health system strengthening and disease-specific initiatives. Country ownership is key to achieving the IA2030 vision because the most important actions will be the responsibility of individual countries.
• Leveraging and strengthening existing mechanisms for coordination, accountability, planning, M&E and advocacy at country, regional and global levels.
• Promoting continuous quality improvement cycles using timely, reliable and fit-for-purpose data.
• Building and strengthening stakeholder accountability and technical alignment to address country needs.
• Aligning and harmonizing with existing regional and national plans and global strategies, including the Sustainable Development Goals (SDGs), Universal Health Coverage (UHC) and Gavi 5.0.

## 2 IA2030 Framework for action

**Fig. 1:**
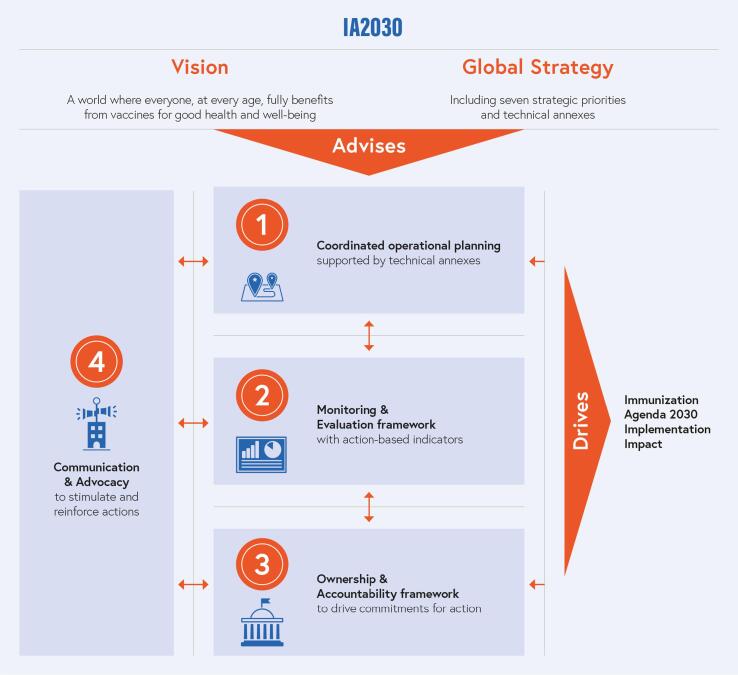
IA2030 Framework for Action with four operational elements to drive implementation.

Four key operational elements are integrated to empower and drive actions to advance the implementation of IA2030 (Fig. 1).

Each of these elements is critical for continuous quality improvement of immunization programmes and other progress required to achieve the IA2030 vision:

1. Coordinated Operational Planning with prioritized actions for implementation by countries, regions and partners, and supported by guidance provided in technical annexes for each of the seven IA2030 strategic priorities.
2. Monitoring & Evaluation (M&E) with action-based indicators to monitor and evaluate progress toward IA2030 goals and strategic priority objectives, to inform corrective actions when needed.
3. Ownership & Accountability (O&A) with structures and platforms to ensure commitments by stakeholders are captured, technical support is facilitated and aligned, and progress is tracked.
4. Communication and Advocacy (C&A), a cross-cutting enabler that will drive coordinated messaging and action at key moments to deliver on accountability objectives throughout the decade.

### 2.1 Coordinated operational planning

Coordinated operational planning by Member States, regional bodies, development partners and civil society is the means to translate the vision of IA2030 into concrete, near-term actions. Taking into account national context and expertise, Member States will incorporate prioritized aspects of IA2030 into their national strategies and plans as they are updated. Initial priorities will include scaling up of COVID-19 vaccination and recovery of immunization and other essential health services to at least pre-COVID-19 levels.

IA2030 operational planning is fully coordinated with existing mechanisms (such as RITAGs and NITAGs) used by regions and Member States as they set regional and national immunization priorities, and develop implementation plans to achieve health-related SDG targets. It will also take into account timebound initiatives (e.g., COVAX), complement Gavi’s 2021–2025 strategy, incorporate learning from the period of the GVAP and seek integration of disease-specific initiatives. While planning processes varied across countries and regions, they have incorporated similar key steps to ensure that immunization needs are fully understood, gaps are covered, prioritization is locally relevant and realistic and meaningful targets are set, and sufficient resources are committed.

Key planning steps include assembling relevant stakeholders from within and beyond immunization and health to review evidence and lessons learned, to understand root causes and to identify improvement needs. Planning processes refers to best practice and draw on up-to-date technical guidance (such as that provided in the IA2030 technical annexes). To support country planning, WHO has released updated guidelines to countries on **developing national immunization strategies,** which incorporate salient points and key shifts in IA2030^5^. It will also be important for CSOs and development partners to align their contributions to achieving IA2030 goals and targets.

IA2030 operational planning will also reinforce alignment and integration across initiatives to control, eliminate and eradicate specific diseases, such as those for polio and measles and rubella. In defining its new endgame strategy, the Global Polio Eradication Initiative (GPEI)^6^ articulates its commitments to IA2030 and demonstrates how the integration of polio eradication and essential immunization activities will contribute to IA2030 strategic priorities. Similarly, the Measles & Rubella Initiative’s new ten-year strategic framework^7^ explicitly identifies contributions to each IA2030 strategic priority, facilitating integration into national and regional planning processes.

### 2.2 Monitoring & Evaluation

The IA2030 Monitoring & Evaluation (M&E) Framework builds on the lessons learned from the Global Vaccine Action Plan (GVAP)^8^ and rather than just being a tool for measuring global progress, it proposes action-based indicators to enable feedback loops at country, regional and global level, and the more extensive use of data at national and subnational levels.

ME&A cycles, facilitated through regular independent technical review at country, regional and global levels, encourage immunization programme stakeholders to continuously ask the questions:

• How are we doing? (Monitor)
• How can we do it better? (Evaluate)
• Who is responsible, for doing what, to make improvements? (Act)

**Fig. 2:**
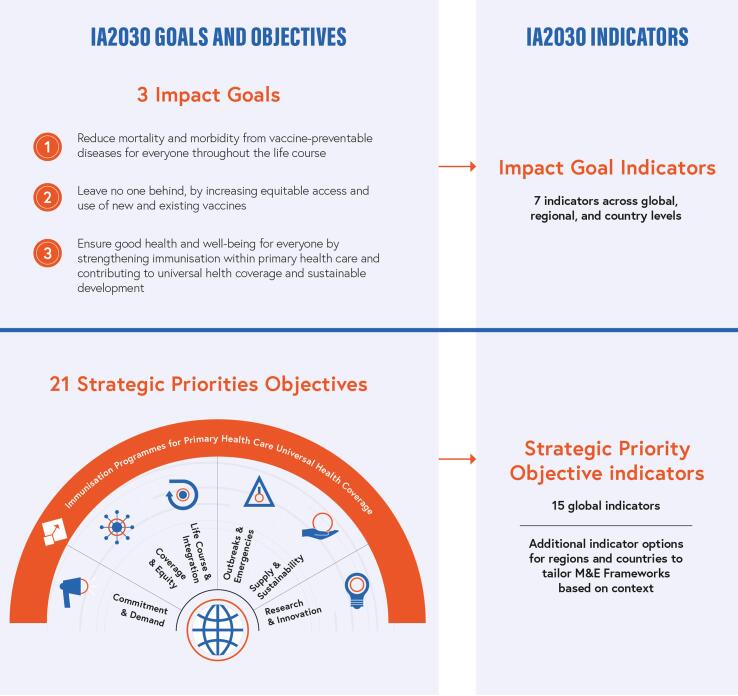
IA2030 Goals, Objectives, and Indicators.

The M&E Framework includes tailored indicators to enable the use of data for action to continuously improve immunization programmes at all levels. It provides indicators to monitor progress towards the three IA2030 impact goals and the 21 objectives within its seven strategic priority areas (Fig. 2).

There are seven impact goal indicators (Table 1). They are outcome and impact measures common across all levels (country, regional and global) and designed to track progress toward the three IA2030 impact goals. Progress made in achieving the impact goal indicators will be assessed against predetermined targets. A detailed description of each impact goal indicator, including target-setting methods and key uses of the indicator for monitoring, evaluation and action, is provided in Annex 1.

**Table 1:** Proposed IA2030 Impact Goal Indicators and Targets.

Impact Goal		Indicator	2030 Target
1 Prevent disease	Save lives	1.1 Number of future deaths averted through immunization^1^	50 million future deaths averted globally
	Control, eliminate & eradicate VPDs	1.2 Number and % of countries achieving endorsed regional or global VPD control, elimination and eradication targets^2^	All countries achieve the endorsed regional or global VPD control, elimination and eradication targets
	Reduce VPD outbreaks	1.3 Number of large or disruptive VPD outbreaks^3^	All selected VPDs^3^ have a declining trend in the global annual number of large or disruptive outbreaks

2 Promote Equity	Leave no one behind	2.1 Number of zero dose children	50% reduction in the number of zero dose children at country, regional, and global levels
	Provide access to all vaccines	2.2 Introduction of new or under-utilized vaccines^4^ in low- and middle-income countries	500 vaccine introductions

3 Build strong immunization programmes	Deliver across the life course	3.1 Vaccination coverage across the life course (DTP3, MCV2, PCV3, HPVc)^5^	90% global coverage for DTP3, MCV2, PCV3, and HPVc
	Contribute to PHC/UHC	3.2 UHC Index of Service Coverage	Improve UHC Index of Service Coverage at country, regional, and global levels

1. Vaccine antigens included: HepB, Hib, HPV, JE, measles, MenA, Streptococcus pneumoniae, rotavirus, rubella, yellow fever, diphtheria, tetanus, pertussis, BCG. Measured relative to zero coverage levels (absence of vaccination); target includes deaths averted over the lifetime of the birth cohort by vaccines given during 2021–30.

2. Eradication (polio), elimination of transmission (measles, rubella), elimination as a public health problem (MNT, hepatitis B), control (Japanese encephalitis)

3. Large or disruptive outbreaks of measles, polio, meningococcus, yellow fever, cholera, and Ebola will be defined based on criteria for each disease.

4. Vaccines included: HepB birth dose, Hib, HPV, IPV2, MCV2, PCV, rotavirus, rubella, DTP booster, COVID-19, JE, YF, MenA, multivalent meningitis, typhoid, cholera, dengue, rabies, HepA, influenza, varicella, and mumps. Malaria and other relevant vaccines will potentially be included when recommended.

Strategic priority objective indicators are designed to track performance towards the 21 IA2030 strategic priority objectives. They will also help to identify potential root causes of success and failure so that actions to improve programme performance can be recommended and implemented. These indicators are a combination of input, process, output and outcome measures, reflecting the need for performance monitoring at country, regional and global levels. Global targets have not been set for strategic priority objective indicators due to wide country and regional variations. Regions and countries are encouraged to assess the baseline for each indicator and to set targets for these indicators that reflect local context.

• **Country strategic priority objective indicators** are intended to be used by country bodies to assess progress, recommend actions for immunization performance improvement, and to inform prioritization and allocation of resources and policy development at facility, sub-national and national levels. To supplement global and regional indicators, WHO and UNICEF Country and Regional Offices are encouraged to support Member States to select additional strategic priority objective indicators for M&E of national health or immunization plans and strategies that are tailored to local needs and context.
• **Regional strategic priority objective indicators** are intended for use by regional bodies to assess progress, recommend actions for performance improvement and to inform tailored technical support to countries.^9^ To supplement global indicators, WHO and UNICEF Regional Offices are encouraged to select additional strategic priority objective indicators that are tailored to regional needs and context.
• **Global strategic priority objective indicators** (n = 15) are intended to assess progress and be used to recommend actions for performance improvement at the global level and to highlight critical performance gaps that need to be further evaluated and tackled at regional and country levels (Table 2). A detailed description of each indicator is provided in Annex 1.

**Table 2:** Proposed IA2030 Global Strategic Priority Objective Indicators (n = 15) (Detailed in Annex 1 in the Framework for action).

**SP 1: Immunization Programmes for PHC/UHC**	1.1 Proportion of countries with evidence of adopted mechanism for monitoring, evaluation and action at national and subnational levels	1.2 Density of physicians, nurses and midwives per 10,000 population	1.3 Proportion of countries with on-time reporting from 90% of districts for suspected cases of all priority VPDs included in nationwide surveillance	1.4 Proportion of time with full availability of DTPcv and MCV at service delivery level (mean across countries)	1.6 Proportion of countries with at least 1 documented individual serious AEFI case safety report per million total population
**SP 2: Commitment & Demand**	2.1 Proportion of countries with legislation in place that is supportive of immunization as a public good		2.2 Proportion of countries that have implemented behavioural or social strategies (i.e., demand generation strategies) to address under-vaccination		
**SP 3: Coverage & Equity**	3.2 DTP3, MCV1, and MCV2 coverage in the 20% of districts with lowest coverage (mean across countries)*				
**SP 4: Life course & Integration**	4.1 Breadth of protection (mean coverage for all WHO-recommended vaccine antigens, by country)				
**SP 5: Outbreaks & Emergencies**	5.1 Proportion of polio, measles, meningococcus, yellow fever, cholera, and Ebola outbreaks** with timely detection and response				
**SP 6: Supply & Sustainability**	6.1 Level of health of the vaccine market, disaggregated by vaccine antigens and country typology**		6.2 Proportion of countries whose domestic government and donor expenditure on primary health care increased or remained stable	6.3 Proportion of countries whose share of national immunization schedule vaccine expenditure funded by domestic government resources increased	
**SP 7: Research & Innovation**	7.1 Proportion of countries with an immunization research agenda		7.2 Progress towards global research and development targets****		

Regional and country specific diseases will be considered in regional and country specific frameworks

^***^Following attributes will be measured: supply meeting demand; individual supplier risk; buffer capacity; long term competition

* Will analyze performance in the bottom 20% with national average

** Includes only outbreaks with an outbreak response vaccination campaign.

**** Targets will be set no later than 2022 and endorsed by SAGE

Through monitoring and analysis of IA2030 indicator progress, independent technical review bodies can recommend areas for further in-depth evaluation to be conducted by national and regional bodies and IA2030 Working Groups, as described in the next section. Evaluation of policies, strategies, and interventions within each strategic priority will be encouraged at country, regional, and global levels as integral to ME&A cycles. Diverse evaluation methods will be needed to assess policies, strategies, and interventions across different contexts. Evaluation efforts conducted by Working Groups would be informed through Consultative Engagement with countries, regions, partners, and civil society, as well as feedback from independent technical review groups (e.g., SAGE, RITAGs) and the global-level IA2030 partnership.

### 2.3 Ownership & accountability

Achieving the vision laid out in the ten-year IA2030 strategy will depend on numerous and varied stakeholders, each taking on agreed responsibilities to achieve the stated goals (ownership). Ensuring these contributions are understood, executed and monitored, a process for checking responsibilities across stakeholders (accountability) will help countries and partners remain on track.

As such, the O&A model for IA2030 makes visible the global, as well as, regional and country commitments made by different stakeholders and ensures accountability by regular monitoring. The global level O&A mechanism is described in the framework for action. The Regional and country level O&A mechanisms will build on existing functioning mechanisms and will be defined further in the Regional and national adaptations of IA2030. Supported by the IA2030 M&E Framework, partners at all levels will have the data to review progress and performance against milestones so that they can take corrective actions when required. Should gaps remain, the O&A model relies on regular partner coordination and oversight by the mechanisms described below to ensure these are adequately addressed.

As highlighted by the UN’s Independent Accountability Panel’s 2020 Report^10^, an effective accountability framework relies on four interconnected pillars, prompting the following questions:

• **Commit:** Have we committed to specific goals, defined responsibilities and required resources?
• **Justify:** Have our decisions and actions to strengthen the achievement of goals and rights been justified by evidence, rights and rule of law?
• **Implement:** Will we monitor and review data, including through independent review, enact remedies, and take necessary action?
• **Progress:** Will we continuously make effective, efficient and equitable progress toward agreed rights and goals?

**Fig. 3:**
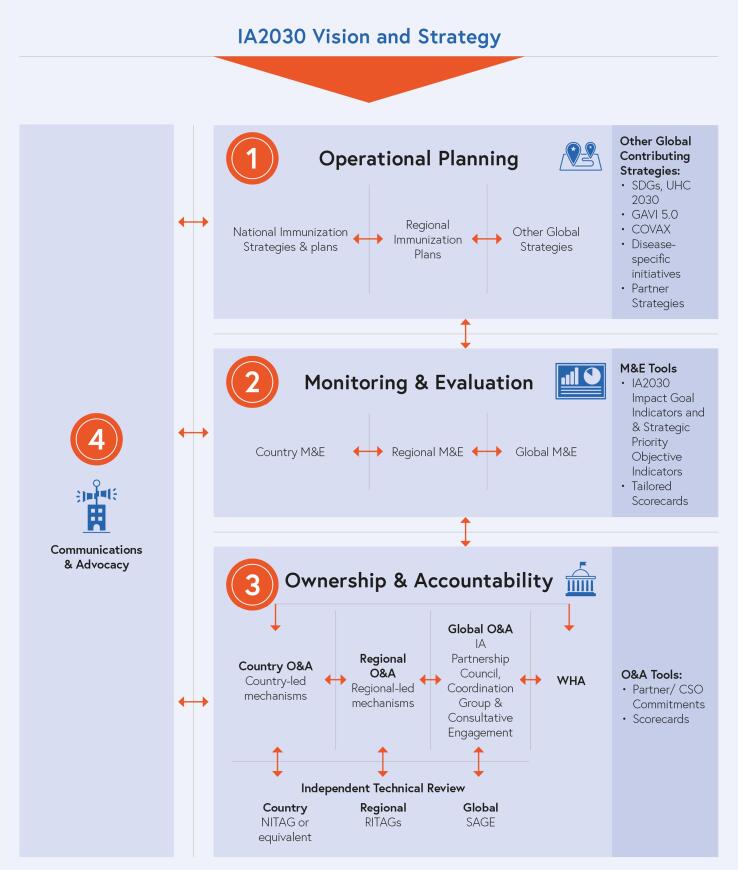
IA2030 Information flow, supported by four operational elements.

This “good practice” framework guides the design of an O&A approach, integrating the necessary structures, tools and information flow (Fig. 3).

In creating the approach to O&A, Member States and development partners have called for more systematic and coordinated use of existing structures across country, regional and global levels. In addition, the shared contributions of development partners (including the private sector) and CSOs should be tailored to specific country and regional contexts, with increased visibility and consolidation of vaccine-preventable disease-specific initiatives.

The global level partnership model for IA2030 provides an overarching ‘umbrella’ forum for immunization intended to represent the interests of all countries, give voice to civil society stakeholders, and cover all vaccine-preventable diseases. It will do so by combining consultative engagement processes through working groups, operational alignment through a Coordination Group, and political leadership through a Partnership Council. It will use newly designed tools to bring greater visibility and evidence to inform decisions across partners to drive corrective actions at country, regional and global levels to achieve the IA2030 vision.

**Fig. 4:**
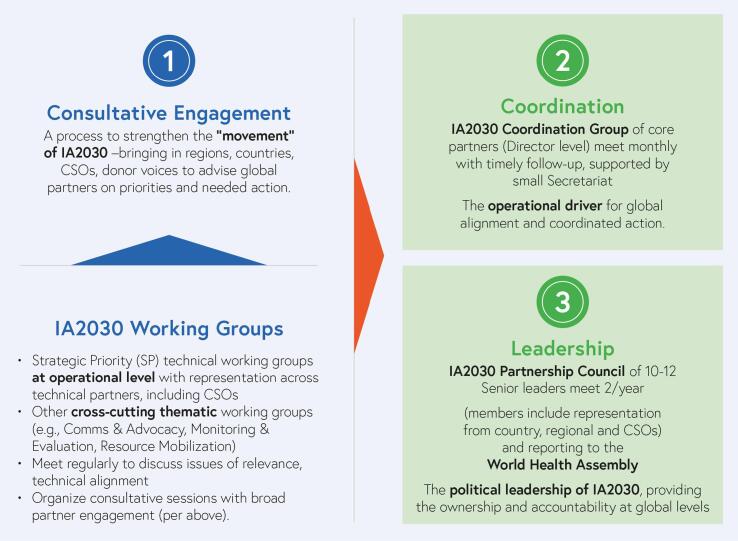
The three components to IA2030 global level O&A model.

As such, the model comprises three interrelated pillars as depicted below (Fig. 4), each playing an important role to form the basis of the IA2030 global partnership. Working together, the components align to the decade’s new vision and strategy. The three components are presented below and are further detailed in an O&A Annex to this Framework.^11^

The following principles will guide the functioning of the global level O&A model:

• **Offers stakeholders something different**: To avoid duplication, the model will maintain a focus on immunization, while also ensuring close engagement with broader health agendas, such as UHC and maternal, neonatal and child health.
• **Gives voice to all countries, regions and communities**: The approach will ensure that all stakeholder groups can engage meaningfully in global-level deliberations.
• **Leverages country and regional structures**: The model will use a variety of existing fora for reviewing development partner, CSO and Member State progress against pledges and targets, as captured in scorecards.
• **Addresses fragmentation:** The approach aims to build consensus and create incentives for partners to work more effectively across disease-specific initatives.
• **Focuses on priorities**: Dialogue at global levels and resulting actions will target priority countries and priority topics as identified through data analytics, consultative processes, and thematic working groups.
• **Keeps a technical focus**: To build on the valuable collaborations used for the development of IA2030 strategic priorities, IA2030 Working Groups will meet routinely to facilitate technical alignment in strategic priority areas, shaping global coordination and actions.
• **Term-limited:** In recognition of the complex and ever-evolving global health landscape, with its myriad of initiatives and numerous partner mechanisms, the model will have a limited term of three years, followed by a full review by the partnership to assess its value and determine its future.

Working Groups were initially organized around the IA2030 strategic priorities to support the collaborative development of the IA2030 vision and strategy (2019) and technical annexes (2020). Working Groups will continue to play an important convening role during 2021–2023 to support focused discussions and technical alignment across thematic or cross-cutting areas of focus, including support for global M&E and C&A. They may complement, extend, incorporate, or be incorporated by existing mechanisms at global or regional levels such as those established for COVAX facility, Gavi 5.0 and/or disease-specific initiatives. Working Groups will shape regular discussions at the operational level, identify areas that require attention by regional or global actors, and feed into the global-level structures, including the Coordination Group and the IA2030 Partnership Council (IAPC) as described below.

Consultative engagement with countries, regions, CSOs and other partners on IA2030 implementation topics will be organized to provide real-time exchange on immunization programme successes and challenges, and to offer peer-to-peer learning and knowledge sharing across sectors and countries. On a rotating basis, and based on topics proposed by countries and regions, Working Groups (or partnership constituencies or communities of practice) will be supported to host open, multilingual “virtual events” with structured format and facilitation to amplify participant contributions. These consultative engagement “touch points” will help identify and elevate issues for consideration by the Coordination Group and IAPC. As such, they contribute to the “movement” of IA2030, bringing in critical voices and perspectives from regions, countries and CSOs in a predictable and structured way and feeding into debates at the global level.

The IA2030 Coordination Group will comprise 7–8 Program Directors from leading immunization agencies and partners. In oversight roles at global levels, these individuals will consider input received through IA2030 Working Groups and consultative engagement, helping drive solutions to address operational bottlenecks and technical alignment. The Coordination Group will also advise on the preparation of formal IA2030 reports (e.g. for WHA, SAGE) and set the agenda for IAPC meetings.

Meeting on a monthly basis, core partners maintain a regular (and more informal) dialogue in support of IA2030 implementation. A small ‘virtual’ IA2030 Secretariat team will be created with dedicated staff from partner organizations to provide logistics and technical support to the IA2030 Coordination Group and associated structures.

The IA2030 Partnership Council (IAPC) comprises 10–12 senior leaders from immunization partners operating at the global level as well as representatives from countries, regions and civil society. The IAPC reinforces, complements and builds upon existing structures at national and regional levels, and focuses global partner attention on priority technical areas, implementation bottlenecks, progress against global immunization targets and partner commitments. It has been created as an accountability mechanism (or governance structure) to jump-start the IA2030 decade with three key objectives:

• Monitor and review progress against IA2030 targets and global partner support
• Advocate for, invest in and align identified key actions to enhance progress
• Mobilize political leadership and drive global partner action

Domestic financing will remain the most important contribution overall in immunization and still remains inadequate in many countries. Development partners and CSOs will specify their intended commitments and additional contributions, aligned to their technical roles and the IA2030 strategic priorities. This will ensure greater transparency and facilitate monitoring of their contributions, and promote accountability for the achievement of IA2030 goals. This process is currently being developed, and is intended to complement and align with existing pledging mechanisms such as Gavi, GPEI and others.

Commitments can take various forms. Some partners could commit financial support, human resources or logistical support (e.g., the management of the IA2030 Secretariat). Others could commit to take the technical lead on specific IA2030 strategic priority areas at the global, regional or country level, or to take on key roles in regional communication and advocacy.

At the global level, development partners and CSOs may map existing commitments to assess gaps and inform resource mobilization efforts. Ultimately, these commitments (current and new) could form part of the Scorecards (see next section) and be made available on the IA2030 website. Each year the IAPC will review progress against these pledges, with updates expected every 3–5 years. At the regional and country level, the frequency of pledging will be adjusted to regional and Member State planning cycles and will take place within existing coordination mechanisms.

Scorecards will be used to track progress as reported through IA2030 impact goal and strategic priority indicator results and pledged commitments for technical resources, advocacy resources, and financial resources. IA2030 scorecards will be used for two distinct objectives:

• To measure progress towards IA2030 impact goal and global strategic priorities and to see contributions made towards these from country, regional and global levels.
• To measure progress against publicly pledged commitments by development partners and CSOs at the global, regional and country levels.

The scorecards will be tailored for use by countries, regions and global-level actors. They will be used to inform decision-making and focus attention on priorities, highlight progress, encourage learning across Member States, support resource mobilization efforts, planning and collaboration and drive corrective action. The tailored approach will support greater accountability of countries, development partners and CSOs.

WHO will facilitate the development of global scorecards annually, compiling data from IA2030 M&E Framework indicators and other sources (e.g. pledges from partners). Scorecards will be reviewed at the global level by the IA2030 Partnership Council and independent technical review bodies. Scorecard templates will be provided to regions and countries to facilitate tailored monitoring, evaluation and action cycles.

### 2.4 Communications & advocacy as a cross-cutting enabler

Communications and Advocacy (C&A) will be essential to underpin coordinated operational planning, Monitoring & Evaluation, and Ownership & Accountability, driving political commitment, country ownership and awareness of IA2030.

The key objectives guiding the development of the C&A strategy are to:

• Ensure immunization remains high on the global health agenda and is integrated with broader themes such as the Sustainable Development Goals, Universal Health Coverage, nutrition and gender.
• Ensure strong ownership of IA2030 by Member States to drive prioritization and progress on immunization.
• Reinforce accountability for progress on immunization goals, and to recognize and celebrate success.

The C&A strategy will develop an approach that is acceptable, both technically and culturally, in different regional and Member State contexts and helps to create a broad social movement for immunization. Language and concepts that are broadly accessible will be used so as to engage with all sectors of the community.

Key messages include the importance of immunization to global health security, its potential to provide the foundation for resilient and sustainable primary health care systems delivering universal health coverage, the importance of access and equity (including reaching zero-dose children), and the role of innovation to enhance the reach and impact of immunization programmes.

A key to success for IA2030 will be ensuring ongoing partner participation and a sustained commitment to the shared vision. Therefore, central to C&A operationalization will be the creation of structures and activities to maintain momentum beyond the launch. A key aim will be to mobilize stakeholders regularly around important milestones and crucial moments, creating a drumbeat of activities throughout the decade. This will ensure that immunization remains high on global and regional health agendas, and help to generate a groundswell of support or social movement for immunization. C&A will collaborate closely with IA2030 Working Groups to align on priorities, identify engagement opportunities, coordinate action and strengthen accountability for IA2030 targets, and celebrate progress.

Flexible, adaptable initiatives, tailored to a range of audiences, will also help regions and Member States to contextualize data and evidence, and advance messages across a variety of platforms. The C&A strategy will align with the work of other communication initiatives to promote confidence in, and demand for, vaccines.

## 3 IA2030 implementation by level

The IA2030 Framework for Action will be taken forward at country, regional and global levels, supported by the following key tools, structures and processes.

### 3.1 Country-level implementation

Member States are ultimately responsible for implementing and financing IA2030 through concrete, national plans and budgets, including those focused on COVID-19 vaccine implementation and recovery of essential health services during the initial years of IA2030. Country commitments are critical to achieve and sustain national immunization targets and goals contributing to the shared IA2030 vision.

Member States will prioritize elements of IA2030 according to their national and regional contexts. For example, many are likely to prioritize concrete, national plans focused on COVID-19 vaccine implementation and recovery of essential health services initially. Some countries with high coverage and well-resourced programmes may focus primarily on rebutting efforts to undermine confidence in vaccines on social media platforms. Other countries may also prioritize access to affordable, quality-assured vaccine supplies or strategies to target children being missed by integrated health services. Introductions of recommended vaccines not yet included in immunization programmes may be a primary priority for other countries. Each country working to address its respective priorities within IA2030 will contribute to achieving shared global impact.

Member State implementation of IA2030 through their respective national strategies and plans (Table 3) will build upon:

• **Technical input from experts:** Tailored country support, coordinated through WHO and UNICEF regional offices, and leveraging national and regional technical advisory groups (e.g. NITAGs, RITAGs) will build upon guidance from SAGE to help ministries of health prioritize. Technical annexes for each IA2030 Strategic Priority will help Member States to identify actions to address programmatic priorities.
• **Updated national immunization strategies and operational plans:** As current plans expire, Member States will update national strategies and operational plans reflecting their emerging priorities in the context of COVID-19 response and recovery and longer-term IA2030 goals.
• **Monitoring, evaluation and action (ME&A) cycles:** Member States will be encouraged to implement ME&A cycles (including effective feedback loops) at all levels to: (1) measure and review IA2030 impact goal and strategic priority objective indicator data on a yearly basis; (2) assess national/subnational and partner/CSO progress using tailored indicator scorecards or dashboards, identify potential root causes of success and failure, and identify areas for improvement; and (3) recommend, plan, implement and review actions to improve programme performance. These cycles will need to take into account the impact of COVID-19, such as when estimating baseline immunization coverage.
• **Strengthened, tangible contributions of different in-country stakeholders:** Some countries may establish formal national accountability frameworks or build on independent health observatories that monitor progress on UHC. Other countries may build on existing and strengthened mechanisms such as inter-agency or health sector coordinating committees (ICCs, HSCCs), NITAGs or the Gavi Alliance Joint Appraisal process. Whether through new or existing platforms, partners will need renewed focus on holding each other accountable. This increased accountability for contributions across in-country partners will support more effective and coordinated implementation of national priorities. CSOs play a growing role, for example connecting national strategies to communities, to strengthen confidence in immunization and to identify marginalized populations with low immunization rates. Countries are encouraged to include CSOs in accountability mechanisms.

**Table 3:** Country Implementation of IA2030.

**Commitment**	**To achieve and sustain national and regional immunization goals & targets**	
**Differentiated IA2030 Priorities**	**According to country context (e.g., coverage & equity, hesitancy, integration of services, outbreaks, quality assured vaccine supply, sustainability)**	
**Advocacy & Communications**	**National communication and advocacy platforms**	
**Coordinated Operational Planning**	**Monitoring & Evaluation**	**Ownership & Accountability**
**Tools & Structures**		
• National Health Strategy • National Immunization Strategy • Prioritized operational plans informed by experts (e.g., NITAGs, RITAGs, SAGE)	• IA2030 IG indicators, Global and Regional SP Objective indicators, and additional SP Objective indicators selected by countries tailored to needs and context • Scorecards or dashboards to measure national/subnational & partner/CSO progress • Monitoring frameworks (e.g., National Health Observatory; WHO-UNICEF JRF)	• WHA representation • Regional Committee representation • NITAGs • ICCs/ HSCCs • Civil Society platforms

**Processes**		
• Coordination through country structures with inclusion of CSOs (e.g., Stakeholder engagement groups, Gavi Joint Appraisal process, Health Sector Coordinating Committee)	Monitoring, evaluation and action cycles (including effective feedback loops) at all levels: • Monitor: measure and review IA2030 indicator data on a regular basis • Evaluate: assess progress using tailored indicator scorecards and identify potential root causes of success and failure • Act: recommend actions for implementation, resource allocation and policy development	• Processes to increase accountability of government, partners & CSOs (e.g., Joint Appraisal in Gavi countries, National Accountability Frameworks) • Routine opportunities for consultative engagement organized by Working Groups

### 3.2 Regional collaboration and support

Member States, development partners and civil society will work together to advance coordinated IA2030 implementation through regional technical and political fora. The initial priority in many regions is likely to be COVID-19 vaccine implementation, and recovery of immunization and essential services to pre-COVID-19 baseline. Regions will need to tailor regional operational plans to emerging priorities arising after COVID-19 recovery is underway, and drive results to ensure that country programmes meet longer-term regional goals and targets aligned to IA2030. Communication and advocacy focal points will contribute to generating and maintaining support for immunization and IA2030′s goals. Views from across regions will be amplified through the consultative engagement process to inform and help hold accountable global level coordination and leadership processes.

Regional cooperation and support (Table 4) will be implemented by:

• **Tailoring IA2030 strategic priorities to regional priorities:** Regional public health experts (e.g., RITAGs facilitated by development partners) will recommend key technical areas for focus across Member States and means to strengthen integration of immunization, including disease-specific initiatives, within UHC/PHC. Regional priorities will be reflected in strategies, operational plans and M&E frameworks, contributing to global impact goals. They will include considerations of changes in approaches necessary where progress has plateaued and in light of targets endorsed by regional and global bodies. Regional structures such as RITAGs will assist Member States, development partners and CSOs to regularly monitor progress and systematically identify emerging priorities.
• **Member States determining regional priorities:** Member states will review and decide on the recommendations from various regional structures (e.g., RITAGs) through Regional Committees, including responses to pandemic and epidemic-prone diseases with the potential for region-wide impact.
• **Monitoring, evaluation and action (ME&A) cycles:** Regions will also implement their ME&A cycles to: (1) measure and review IA2030 indicator data from countries on a yearly basis, with a more extensive evaluation at the mid-point (2025) and end-point (2030) of the IA2030; (2) assess regional/national and partner/CSO progress using tailored indicator scorecards, identify potential root causes of success and failure, and identify areas for improvement; and (3) recommend actions for improvement of regional performance and identify technical support needed for countries to plan and implement actions to improve programme performance. These cycles will need to take into account the impact of COVID-19, such as when estimating baseline immunization coverage.
• **Development partner coordination:** Regional priorities will be reflected in regional operational plans with key focus areas for support across Member States. Initial plans are likely to include a stock-taking timepoint as countries emerge from the COVID-19 pandemic, allowing for regions to reset priorities. Development partners will pledge their commitments (e.g., support for specific technical functions) for IA2030, contributing to coordinated support to Member States and promoting greater accountability. Strengthened Regional Interagency Coordinating Committees (RICCs) can align development partner strategies to regional IA2030 priorities. Regional working groups (RWGs) coordinating development partner operational support to countries can be strengthened, with expanded remits and more systematic inclusion of CSOs.
• **CSO commitments:** CSOs will increase the transparency of commitments, roles and contributions to immunization. They will reflect their commitments in pledges.
• **Shared commitments through regional political and economic mechanisms:** Member States will guide the process of seeking commitments and monitoring progress through mechanisms at regional (e.g., African Union, European Union, Association of South-East Asian Nations) or sub-regional (e.g., Southern African Development Community) levels. Political commitments will complement technical commitments and mobilize the support of wider ownership and accountability by partners beyond immunization and health.

**Table 4:** Regional Implementation of IA2030.

Commitment	To achieve and sustain national and regional immunization goals & targets	
Differentiated IA2030 Priorities	According to country context (e.g., coverage & equity, hesitancy, integration of services, outbreaks, quality assured vaccine supply, sustainability)	
Advocacy & Communications	Regional communication and advocacy platforms	
Coordinated Operational Planning	Monitoring & Evaluation	Ownership & Accountability
**Tools & Structures**		
• Regional IA2030 Plans • 3–5 year regional operational plans • Regional Working Groups (e.g., strengthening of existing Gavi groups to include CSOs and coordinate support to non-Gavi countries) • Regional Interagency Coordinating Committees	• IA2030 Impact Goal indicators, Global and Regional SP Objective indicators, and additional SP Objective indicators selected by regions tailored to needs and context • Scorecards with country and regional progress • Scorecards for partner/CSO progress • WHO-UNICEF Joint Reporting Form • WHO Immunization Information System	• RITAGs • Regional Committees • Regional Working Groups • Other Regionally tailored structures (e.g., Regional Cooperation Organizations, Regional Accountability Councils)

**Processes**		
• RITAGs facilitated by development partners recommend key technical areas for focus across Member States • Coordination with UHC and PHC • Coordination with disease-specific initiatives	• Monitor: compile country data to report on indicators • Evaluate: assess regional/national & partner/CSO progress using tailored indicator scorecards and identify potential root causes of success and failure • Act: Recommend actions for regional perfomance improvement and identify technical support needed for countries	• Multi-year pledges from Partners/CSOs • Routine opportunities for consultative engagement organized by Working Groups

### 3.3 Global commitments

As presented above in Section 2 on O&A, Member States, development partners and civil society will work together at the global level to ensure the highest level of financial, technical and political commitment to IA2030. They will also coordinate responses in priority areas with a global reach, such as advocacy, vaccine supply, innovation and technical guidance. Initial commitments will prioritize COVID-19 vaccine implementation (e.g. through COVAX and Gavi), as well as supporting efforts to re-establish routine immunization and essential services to pre-COVID-19 baseline levels through 2022 and 2023.Table 5

**Table 5:** Global Commitments to IA2030.

**Commitment**	**To sustain the highest level of technical and financial commitment to IA2030**	
**Differentiated IA2030 Priorities**	**According to global function (e.g., coordination, vaccine supply, normative guidance, research & innovation, financing)**	
**Advocacy and communication**	**Global Communication and Advocacy Focal Points**	
**Coordinated Operational Planning**	**Monitoring & Evaluation**	**Ownership & Accountability**
**Tools & Structures**		
• IA Partnership Council (IAPC) • IA2030 Working Groups • Disease-specific strategies and road maps (e.g., GPEI, MRI) • Other Global and Contributing Strategies (e.g., SDGs, UHC 2030, Gavi 5.0, COVAX, partner strategies)	• IA2030 IG indicators and Global SP Objective indicators • WHO-UNICEF JRF • WHO Immunization Information System • Scorecards with country and regional progress • Scorecards for partner/CSO progress	• IA Partnership Council (IAPC) • Coordination Group • WHO Strategic Advisory Group of Experts • World Health Assembly

**Processes**		
Operational plans by topics or SP as need arises	• Monitor: country and global data on IG and SP indicators; compile partner/CSO data to report on progress • Evaluate: assess progress using scorecards and identify potential root causes of success and failure • Act: for performance improvement at global level	• Multi-year pledges from Partners/CSOs • Routine opportunities for consultative engagement organized by Working Groups

In addition, global partners and CSOs will be encouraged to implement regular ME&A cycles to: (1) monitor IA2030 indicator data from countries and regions on a regular basis; (2) evaluate progress to achieve Impact Goals and Strategic Priority Objectives, including independent technical review by SAGE, and (3) identify actions for performance improvement at the global level, and performance gaps to address at regional and country levels. Independent review by SAGE will include: a) assessing regional/national and partner/CSO progress using tailored indicator scorecards, and b) recommending actions for performance improvement, and areas for further evaluation by working groups and disease-specific initiatives to identify root causes of success and failure. ME&A cycles will need to take into account the impact of COVID-19 when estimating baseline for progress measurement.

## 4 IA2030 in the context of covid-19

The COVID-19 pandemic has starkly illustrated the strengths and fragilities of immunization programmes. It has re-emphasized the value of immunization and the need for a flexible and sustainable approach to build country, regional and global immunization capacity.

COVID-19 vaccines, some based on innovative new technological platforms, were developed, evaluated and licensed at unprecedented speed. Valuable lessons can be learned from this experience to accelerate vaccine research and development (R&D) for other infectious diseases for which vaccines are not yet available.

As discussed in previous sections, IA2030 was developed to anticipate pandemics and regional outbreaks while maintaining a focus on progressive improvement in immunization programmes over a decade. In addition to embedding COVID-19 vaccine implementation and recovery throughout planning processes, the IA2030 Strategy’s technical annexes^12^ provide guidance that can be applied to COVID-19 responses, such as:

• **Outbreaks & Emergencies (SP5):** Guidance on the immediate responses needed, including aspects of surveillance, maintaining immunization and other primary health care services, and engaging communities.
• **Vaccine Supply & Sustainability (SP6):** Guidance on the innovative incentives needed to engage manufacturers to develop products for an emerging pathogen.
• **Commitment and Demand (SP2):** Guidance on how to maintain political commitment beyond COVID-19 vaccines, and how to maintain trust and demand for vaccines at all ages.
• **Coverage and Equity (SP3):** Guidance on how to reach all intended target groups for vaccination, including vulnerable communities and those in conflict-affected settings.
• **Research & Innovation (SP7):** Guidance on implementation and operational research supporting immunization services in the context of emerging challenges.

Guidance is also provided on re-building services and ongoing prevention:

• **Immunization within PHC/UHC (SP1):** Guidance on vaccine safety monitoring, supply chain and logistics, and availability of a skilled health workforce as well as recovery through an integrated PHC approach.
• **Life Course & Integration (SP4):** Guidance on implementation of vaccination strategies for older age groups, including adults, with COVID-19 vaccine introduction providing an opportunity to establish and strengthen vaccine platforms for older age groups.

In particular, COVID-19 is impacting approaches to regional and country planning, given that the future course of the pandemic is uncertain. Priority is on near-term, two- or three-year plans for implementing COVID-19 vaccines and re-building of essential services. As the course of recovery becomes more clear, regions and countries will update plans, in consultation with technical experts and regional organizations.

COVID-19 is also likely to impact the development of M&E Frameworks by countries and regions. For example, baseline data and targets are likely to need adjustment, and additional indicators might be needed as more is learned about the impact of COVID-19 on services and how quickly services recover.

More positively, the COVID-19 vaccine deployment and response efforts currently underway across the globe are valuable opportunities to further strengthen the economic case for equitable immunization programmes and to stress the importance of multilateral coordination to global recovery.

## 5 Learning agenda for the path ahead

IA2030 is a living and evolving strategy for the acute COVID-19 response years and the rest of the decade ahead. Member States, development partners and CSOs will need to build from the initial operationalization outlined in this document to address emerging challenges and contextual changes. Mechanisms will need to be created (for example with support from the IA Partnership Council) to capture learning and associated recommendations.

In particular, the IA2030 M&E Framework should remain fit for purpose for the new decade. Thus, the Framework should be reviewed and updated at least once every three years in response to changing needs and improvements in M&E methods to ensure it delivers the data required to improve programme performance. Similarly, the IA2030 technical annexes will also require regular updates over the decade. This need for flexibility is highlighted by the uncertainty associated with recovery from the COVID-19 pandemic and the implementation of COVID-19 vaccines.

An initial set of core questions and topics have been identified for the IA2030 Learning Agenda and are provided below for each operational element.

Ownership & Accountability

• The implications of changing political and financial commitments to immunization, and IA2030 more broadly, in the context of COVID-19 and implementation of COVID-19 vaccines.
• The most efficient means to engage diverse CSOs to strengthen community-level ownership and accountability for immunization.
• The added value of strengthened fora (e.g., Regional Working Groups) or new mechanisms (e.g., IA2030 Partnership Council) and tools designed to secure and sustain stronger ownership and improve accountability (e.g., public pledges and tailored scorecards).
• A review of O&A mechanisms after three years (2023) to identify the need for course corrections.

Operational Planning

• Reviews of how country and regional plans shift during the course of the COVID-19 pandemic and as its influence begins to recede.
• Planning and review processes that extend beyond the traditional WHO/UN mechanisms and engage diverse development partners and CSOs.
• Opportunities for more efficient, timely and reliable data collection and use through digital innovations.

Monitoring & Evaluation

• Review potential means to strengthening capacity at country, regional and global levels to implement ME&A cycles with effective feedback loops.
• Identify means to strengthening both the quality and the use of data for M&E Framework indicators
• Further development of Impact Goal and Strategic Priority Objective indicators and identification of additional indicators needed to identify and track severe gaps in health system performance (see Annex 1).
• Consider linkages with existing monitoring processes and data sources to IA2030 ME&A cycles, including use of the WHO Immunization Information System (WIISE). Efforts should be made to identify owners and actions for all IA2030 indicators and to decrease the data-reporting burden for countries.

Communication & Advocacy

• Responsiveness to changing attitudes around immunization and adaptation of strategies as appropriate.
• Ways to solicit and secure greater community-driven commitment to immunization through CSOs and the subsequent translation into increased national and regional commitments.
• Means to respond to misinformation about vaccines disseminated through changing social media platforms and other ways mis- and dis-information are spread.

## CRediT authorship contribution statement

**Ann Lindstrand** has Conceptualized, Funding acuisition, Methodology, Supervision, Validation, Writing - in both original draft and review and editing.

## Declaration of Competing Interest

The authors declare that they have no known competing financial interests or personal relationships that could have appeared to influence the work reported in this paper.

## Footnotes

Appendix A Supplementary data to this article can be found online at https://doi.org/10.1016/j.vaccine.2021.09.045.

## Footnotes

4 IA2030 Core Team has been co-led by WHO and UNICEF, with members from the Wellcome Trust, Bill & Melinda Gates Foundation, the Gavi Secretariat, US Centers for Disease Control and Prevention, and the Gavi Civil Society Organisation (CSO) Constituency.

9 Guidance for selection of regional and country strategic priority objective indicators is provided in Annex 1.

## Footnotes

1 For the full IA2030 Framework for Action and annexes please go to: http://www.immunizationagenda2030.org/framework-for-action

2 Consultations with WHO Member States took place in December 2020 and April 2021.

3 https://www.who.int/publications/i/item/the-global-vaccine-action-plan-2011–2020-review-and-lessons-learned-strategic-advisory-group-of-experts-on-immunization

5 See https://www.who.int/teams/immunization-vaccines-and-biologicals/vaccine-access/planning-and-financing/nis.

6 Polio Eradication Strategy 2022–2026: Delivering on a Promise, available at: https://polioeradication.org/gpei-strategy-2022–2026/

7 Available at: https://measlesrubellainitiative.org/learn/the-solution/the-strategy/

8 MacDonald et al. Global vaccine action plan lessons learned I: Recommendations for the next decade, Vaccine, Volume 38, Issue 33, 2020, Pages 5364–5371,Vaccine 2020; 38: 5364–71

10 UN Secretary-General’s Independent Accountability Panel (IAP) for Every Woman Every Child. 2020 Report. Geneva: World Health Organization; 2020

11 For the full IA2030 Framework for Action and annexes please go to: http://www.immunizationagenda2030.org/framework-for-action

12 Available on the IA2030 website: http://www.immunizationagenda2030.org

