# Supplementary material for: Implementing the immunization agenda 2030: A framework for action through coordinated planning, monitoring & evaluation, ownership & accountability, and communications & advocacy
Source: Vaccine. 2024 Apr 8;42(Suppl 1):S15–27. doi: 10.1016/j.vaccine.2021.09.045 (PMC10801759; doi:10.1016/j.vaccine.2021.09.045)
Supplement: Supplementary data 1 [file mmc1.docx]

**Annex 1: IA2030 Monitoring and Evaluation Framework**

*The purpose of this document is to describe the Monitoring & Evaluation (M&E) Framework- one of the essential pillars needed to implement the IA2030 Strategy.*

*The M&E Framework provides action-based indicators to monitor and evaluate progress toward IA2030 goals and strategic priority objectives.*

*For more information on the operational elements of IA2030, including operational planning through regional and national strategies, mechanisms to ensure ownership and accountability, and communication and advocacy to stimulate and reinforce required actions by all stakeholders throughout the decade, please see* ***“Implementing the Immunization Agenda 2030: A Framework for Action”*** *available on the IA2030 website: http://www.immunizationagenda2030.org*

# **Background and overview**

The M&E Framework includes **tailored indicators** to enable the use of data for action to continuously improve immunization programmes at all levels. It provides indicators to monitor progress towards the three IA2030 impact goals and the 21 objectives within its seven strategic priority areas (**Figure 1**).

**Figure 1: IA2030 Goals, Objectives, and Indicators**


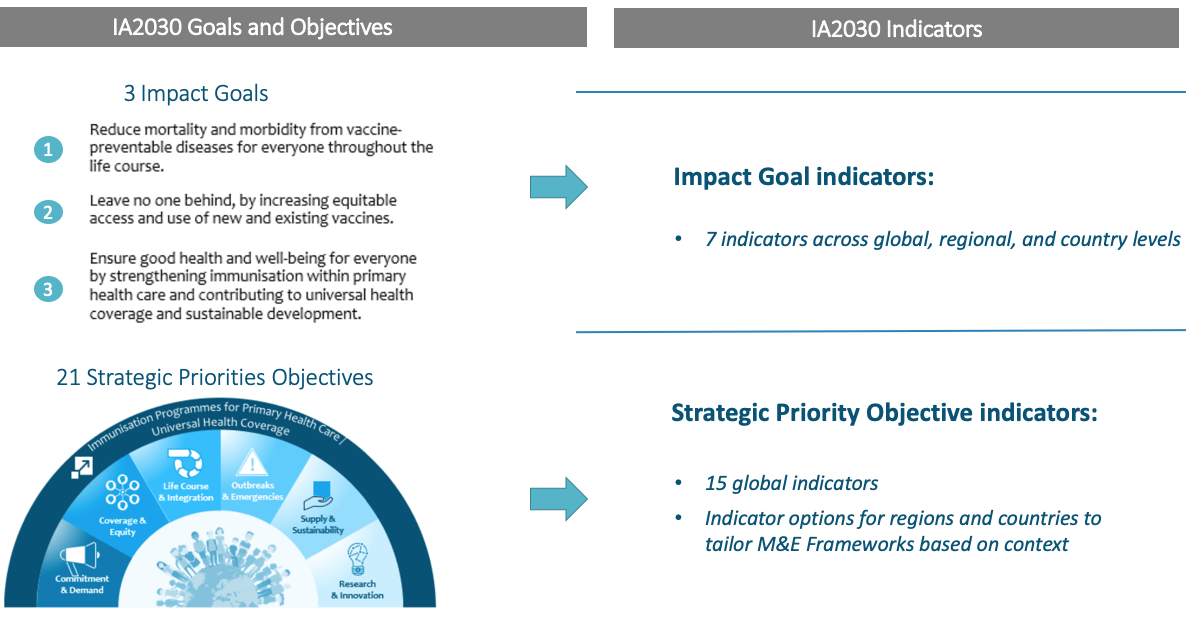


# **Impact Goal Indicators**

There are seven impact goal indicators (**Table 1**). They are outcome and impact measures common across all levels (country, regional and global) and designed to track progress toward the three IA2030 impact goals. Progress made in achieving the impact goal indicators will be assessed against predetermined targets. A detailed description of each impact goal indicator, including target-setting methods and key uses of the indicator for monitoring, evaluation and action, is provided below in the section “Impact Goal (IG) Indicators”.

**Table 1: IA2030 Impact Goal Indicators and Targets**^[[1]](#footnote-1)^

**
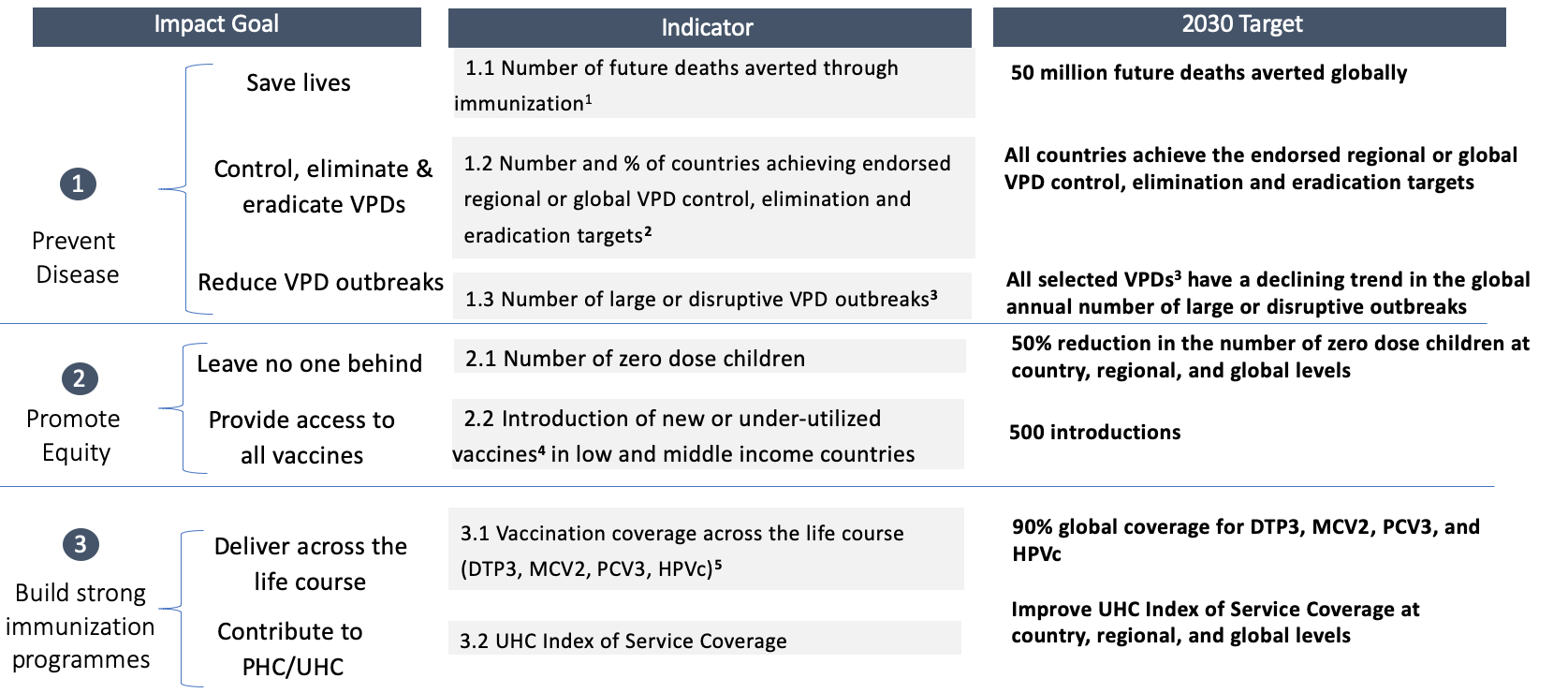
**

# **Strategic Priority Objective Indicators**

Strategic priority objective indicators are designed to track performance towards the 21 IA2030 strategic priority objectives. They will also help to identify potential root causes of success and failure so that actions to improve programme performance can be recommended and implemented. These indicators are a combination of input, process, output and outcome measures, reflecting the need for performance monitoring at country, regional and global levels. Global targets have not been set for strategic priority objective indicators due to wide country and regional variations. Regions and countries are encouraged to assess the baseline for each indicator and to set targets for these indicators that reflect local context.

**Country strategic priority objective indicators** are intended to be used by country bodies to assess progress, recommend actions for immunization performance improvement, and to inform prioritization and allocation of resources and policy development at facility, sub-national and national levels. To supplement global and regional indicators, WHO and UNICEF Country and Regional Offices are encouraged to support Member States to select additional strategic priority objective indicators for M&E of national health or immunization plans and strategies that are tailored to local needs and context.

**Regional strategic priority objective indicators** are intended for use by regional bodies to assess progress, recommend actions for performance improvement and to inform tailored technical support to countries.^[[2]](#footnote-2)^ To supplement global indicators, WHO and UNICEF Regional Offices are encouraged to select additional strategic priority objective indicators that are tailored to regional needs and context.

**Global strategic priority objective indicators (n=15) are intended to assess progress** and be used to recommend actions for performance improvement at the global level and to highlight critical performance gaps that need to be further evaluated and tackled at regional and country levels (Table 2). A detailed description of each indicator is provided below in the section “Strategic Priority Objective Indicators.”

**Table 2: IA2030** **Global Strategic Priority Objective Indicators (n=15)**


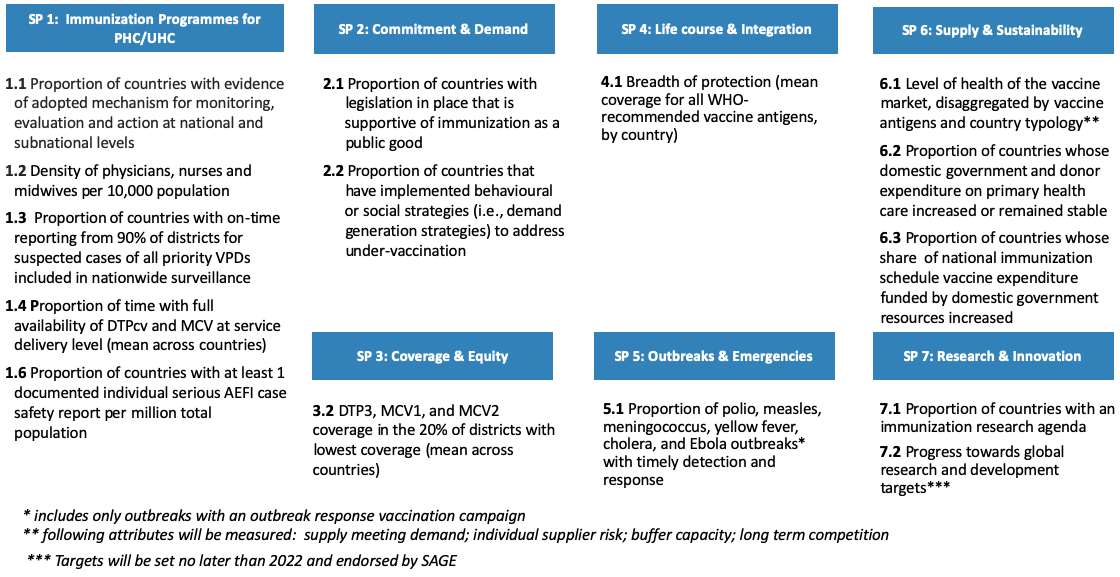


#

**Impact Goal (IG) Indicators**

| **INDICATOR 1.1 Number of future deaths averted through immunization** | | |
| --- | --- | --- |
| **MONITOR**  ***How will progress be monitored?*** | **EVALUATE *How will results of monitoring be evaluated?*** | **ACT**  ***How will evaluation be used for action?*** |
| **Definition:**  Total number of future deaths averted from 2021-2030, based on the IA2030 coverage scenario.  **Measurement approach**:  A modelling approach is used to project the number of deaths averted at the global and regional levels by achieving aspirational coverage targets for IA2030. These targets are also aligned with the Impact Goal indicator 3.1- vaccination coverage across the life course. The initial scope focuses on 14 pathogens, which will be expanded to update the estimates at the midpoint of IA2030.  **2021-2030**: Hepatitis B, Hib, HPV, JE, measles, MenA, *Streptococcus pneumoniae*, rotavirus, rubella, yellow fever, diphtheria, tetanus, pertussis, TB (BCG)  **By 2025**: Polio, typhoid, influenza, cholera, multivalent meningitis, COVID-19, varicella, dengue, mumps, rabies, hepatitis A, hepatitis E, and other new vaccines.  **Calculation:**   - Observed and averted deaths, collected from multiple data sources, are converted into a single measure of country-, age-, and vaccine-specific relative risk of death conditional upon coverage levels. - The relative-risk model is used to predict deaths averted in all locations and diseases. - Additional calibration step converts the estimates into deaths averted by year of vaccination, which allows for capturing the lifetime effect of vaccination aggregated for the year the vaccines are delivered.   **Data source:**  WHO-UNICEF Immunization Coverage estimates, estimates of deaths averted from Vaccine Impact Modelling Consortium (VIMC), Global Burden of Disease Study, and other model inputs from published literature.  **Stakeholder(s) responsible for measurement:**  WHO IVB and DDI project team, project stakeholder committee (BMGF, CDC, Gavi, IHME, VIMC, VIMC Scientific Advisory Board, IVIR-AC, WHO DDI, WHO IVB)  **Frequency of reporting:**  Twice (starting point and midpoint of IA2030) for target setting. The target will be updated for the midpoint based on the expanded scope of pathogens and model updates. | **Baseline:**  **4.3 million deaths averted per year (2019)**  Total number of deaths averted due to vaccination in 2019 based on the historical WUENIC estimates; 2019 was used as the baseline year, rather than 2020, to capture the pre-COVID-19 trend. The estimates are measured relative to zero coverage level (absence of vaccination).  **Target:**  **Increase to 5.8 million deaths averted in 2030**  **50 million total deaths averted during 2021-2030**  Total number of future deaths averted due to vaccination from 2021-2030 based on the aspirational coverage targets described in impact goal 3.1.  For this purpose, 2030 country-level coverage estimates were calculated based on the achievement of three goals all countries are urged to pursue:  Introduction of any missing recommended vaccines  A reduction in zero-dose children by half, compared with 2019 baseline  Achievement of DTPcv-1 coverage that is consistent with the aforementioned zero-dose reduction and coverage for all other vaccines within a 5% range of DTPcv-1  UNPD population estimates for 2019, and projected estimates for 2030 were used to convert absolute numbers of unvaccinated children to equivalent DTPcv-1 targets.  **Analysis and interpretation:**   - Analysis conducted by the WHO IVB and DDI project team; results displayed on shared dashboard; reported at global and regional level. - Results disaggregated by pathogen and year of vaccination.   **Frequency of evaluation:**  Twice (midpoint and endpoint of IA2030) for monitoring and reporting. The midpoint evaluation will focus on 14 pathogens only, based on the models used for the starting point. The endpoint evaluation will focus on the expanded scope of pathogens based on the updated models from the midpoint. | Global, regional, and country partners can use evaluation findings for advocacy in securing commitment and resources for immunization programmes.  Specific recommendations by vaccine highlighted in evaluation may be used to plan disease-specific interventions at global and regional level. |

| **INDICATOR 1.2 Number and proportion of countries that have achieved regional or global VPD control, elimination, and eradication targets** | | |
| --- | --- | --- |
| **MONITOR**  ***How will progress be monitored?*** | **EVALUATE *How will results of monitoring be evaluated?*** | **ACT**  ***How will evaluation be used for action?*** |
| **Definition:** Achievement of all VPD control, elimination, and eradication targets; endorsed by a global or regional body of WHO Member States, with target dates between 2021 and 2030, and that are based on incidence or prevalence measures.  **Measurement approach:** Two monitoring and evaluation cycles will occur annually. The first is the indicator and revision cycle. WHO Regional Offices will conduct a review to confirm and revise the inclusion criteria for each VPD based on, the global or regional endorsement status, target time frame, and the target type and definition.  The second is the assessment and reporting cycle. Established regional verification and certification commissions, or verification committees, will assess the achievement status of the disease specific VPD target for each country.  **Calculation:** Numerator is the number of countries that met the VPD target, and the denominator is the number of countries with an endorsed VPD target based on incidence or prevalence measures.  **Data source:** Verification, certification, and disease-specific committee reports.  **Stakeholder(s) responsible for measurement:** Verification and certification commissions, and validation committees established by WHO Regional Offices with technical assistance from VPD control, elimination, and eradication initiatives.^[[3]](#footnote-3)^  **Frequency of reporting:** Annual | **Baseline:** Number and proportion of countries that have achieved each VPD control, elimination, and eradication target by the end of 2021.  **Target:**   - All countries achieve the endorsed regional or global VPD control, elimination, and eradication targets.   **Analysis and interpretation:** The achievement status of each VPD control, elimination and eradication target, based on incidence and prevalence measures, will be monitored annually. Progress will be monitored and reported during the decade to identify countries at risk of not achieving the target by the specified target date, and to provide visibility of disease-specific progress and risk for adjacent countries and regions.  Annual monitoring of the indicator confirmation and revision cycle will identify the need for new control, elimination, and eradication goals, or changes to the existing targets. The process will also document possible differences across the regions to provide an opportunity to harmonize the target definitions.  **Frequency of evaluation:** Annual. | Global, regional, and country partners can use evaluation findings for operational planning, and for communication and advocacy to:   - ensure needed support to countries to achieve VPD control, elimination, and eradication initiatives, and      - highlight and reinforce coordination of strategies to link VPD control, elimination and eradication initiatives with health system strengthening initiatives. |

| **INDICATOR 1.3 Number of large or disruptive vaccine-preventable disease outbreaks** | | |
| --- | --- | --- |
| **MONITOR**  ***How will progress be monitored?*** | **EVALUATE *How will results of monitoring be evaluated?*** | **ACT**  ***How will evaluation be used for action?*** |
| **Definition:**  A VPD outbreak* meeting size criteria for large or disruptive outbreaks aligned with global vaccine-preventable disease strategies and at least one criterion from Annex 2 of the International Health Regulations (<https://www.who.int/ihr/annex_2/en/>)  *****including measles, wild poliovirus, circulating vaccine derived poliovirus, meningococcus, yellow fever, cholera, and Ebola, the list could be revised, especially as additional diseases become vaccine preventable.  **Measurement approach**:  Large or disruptive VPD outbreaks are identified using data from specific VPD control programmes and from WHO World Health Emergencies surveillance systems. Different criteria were applied for each disease. For multi-country outbreaks, each country’s portion of the outbreak was assessed separately. The overall indicator will function as a composite combining data across the different diseases.  **Calculation:** A collective count of outbreaks of epidemic prone diseases that meet set size criteria, such as the number of cases or disease incidence.  **Data source:** VPD eradication, elimination, and control programmes and the WHO World Health Emergencies surveillance systems.  **Stakeholder(s) responsible for measurement:** International Coordinating Group for Vaccine Provision, WHO Headquarters and WHO Regional Offices with technical assistance from VPD control, elimination and eradication initiatives^[[4]](#footnote-4)^  **Frequency of reporting:** Annual | **Baseline:** The mean number of large or disruptive VPD outbreaks calculated over three years, 2018-2020.  **Target:**  All (100%) of measles, polio, meningococcus, yellow fever, cholera, and Ebola separately show a declining trend in the global annual number of large outbreaks by end of decade.  **Analysis and interpretation:** The level and trend of the number of large or disruptive outbreaks will be analysed annually.  The directionality of the trend will be measured by calculating the line of best fit for the data points (number of annual outbreaks) over time (from baseline to 2030) and assessing its slope.  Number of outbreaks will be reported separately for each disease.  **Frequency of evaluation:** Annual | Global, regional, and country partners can use evaluation findings for operational planning, and for communication and advocacy to:   - ensure timely availability and strategic allocation of vaccines and supplies, mobilization of trained human resources for outbreak response - ensure capacity of immunization programmes to anticipate, prepare for, detect and rapidly respond to VPD and emerging disease outbreaks - ensure capacity of immunization programmes to establish timely and appropriate immunization service delivery during emergencies and in communities affected by conflict, disaster and humanitarian crisis - ensure vaccine introduction and scale up of coverage to prevent newly emerging VPDs - use measles cases and outbreaks as a tracer to identify weaknesses in immunization programmes, and to guide programmatic planning in identifying and addressing these weaknesses. |

| **INDICATOR 2.1. Number of zero-dose children** | | |
| --- | --- | --- |
| **MONITOR**  ***How will progress be monitored?*** | **EVALUATE *How will results of monitoring be evaluated?*** | **ACT**  ***How will evaluation be used for action?*** |
| **Definition**: Zero-dose children are defined as those that lack access to or are never reached by routine immunization services. They are operationally measured as those who lack a first dose of a DTP-containing vaccine.  **Measurement approach**: This indicator is calculated as the difference between the estimated number of surviving infants and the estimated number of children vaccinated with DTPcv-1.  The number of zero-dose children will be determined at country, regional and global level using WHO and UNICEF estimates of national immunization coverage (WUENIC) and UNPD population estimates of birth cohorts, adjusted for surviving infants.  At the national and subnational level, administrative reporting systems can also be used, together with any in-country survey results and other information sources that can help countries establish estimates for zero-dose children.  **Calculation:** This indicator is calculated as the difference between the estimated number of surviving infants and the estimated number of children vaccinated with DTPcv-1.  **Data source:** WUENIC, UNPD population estimates  **Stakeholder(s) responsible for measurement:** WHO IVB, national immunization programmes  **Frequency of reporting**: Annual at regional and global levels, monthly at national and subnational levels | **Baseline:** 14 million children (2019)  **Target:**Reduction in the number of zero-dose children by 50% (all levels). In countries where DTP1 coverage already reaches 99%, the target is to maintain coverage.  **Analysis and interpretation:** The level and trend of the number of zero-dose children needs to be analysed with an equity lens, aiming to find out where inequalities might point to barriers to immunization across specific populations and geographies. This requires disaggregation by subnational levels and other dimensions (socio-economic, language group, ethnicity) as available.  In this context, the number of zero-dose children needs to be used to identify underserved, undervaccinated  communities.  **Frequency of evaluation:** Annual at global and regional levels. Ideally quarterly at national and subnational levels. | At the global and regional level, the number of zero-dose children by region and country will lead to a prioritization of efforts, and can be used to create accountability for countries that do not reach targets, or backslide from previously attained targets. Furthermore, it can be used to communicate about immunization gaps that exist in the world, and advocate for concerted efforts to bridge them.  At the country and subnational level, identifying zero-dose children and underserved communities should facilitate a root-cause analysis of the reasons for under- vaccination, and identification of the barriers that exist for certain communities and geographies. From a communication perspective, the importance of this indicator will highlight the need to focus on equity in immunization. |

| **INDICATOR 2.2 Introduction of new or under-utilized vaccines in low- and middle-income countries** | | |
| --- | --- | --- |
| **MONITOR**  ***How will progress be monitored?*** | **EVALUATE *How will results of monitoring be evaluated?*** | **ACT**  ***How will evaluation be used for action?*** |
| **Definition**:  Introduction* of new or under-utilized vaccines† in low- and middle-income countries.  *Addition of a vaccine to the national immunization schedule and use of the vaccine for a sustained period of at least 12 months (excludes vaccines used only in the private sector that are not in national immunization schedule; includes vaccines in national immunization schedules that are used in at risk populations, e.g., seasonal influenza).  †New or underutilized vaccines are vaccines that have not yet been introduced into national immunization schedules in all countries where recommended by WHO.  **Measurement approach**: Vaccines included in this indicator that are recommended by WHO for use in national immunization schedules in all countries: HepB birth dose, Hib, HPV, IPV2, MCV2, PCV, rotavirus, rubella, DTP booster, and COVID-19 (interim recommendation).  Vaccines included in this indicator that are recommended by WHO for use in national immunization schedules in countries in certain geographic region(s), in some high-risk populations, or in immunization programmes with certain characteristics: YF, JE, MenA, multivalent meningitis, typhoid, cholera, dengue, rabies, HepA, influenza, varicella, and mumps.  Other relevant vaccines (e.g., malaria) will be included when recommended.  Low- and middle-income countries are defined according to the World Bank’s income classifications.  **Calculation:** Count of the number of country vaccine introductions for WHO recommended vaccines reported from 2021-2030.  **Data source:** WHO-UNICEF Joint Reporting Form (JRF)  **Stakeholder(s) responsible for measurement:**  WHO IVB, national immunization programmes  **Frequency of reporting:** Annual | **Baseline:** The number of remaining globally and regionally recommended vaccine introductions as of December 2020 in LMIC was 548. LMIC collectively introduced 519 vaccines in national schedules between 2011 and 2020.  **Target**:  At least 500 vaccine introductions in low- and middle-income countries by 2030 (including current NUVI as well as vaccines that will be recommended over the decade).  **Analysis and interpretation:**  Achievement of introduction of new or underutilized vaccines will be monitored annually.  Analysis and visualization of the indicator will focus on equitable access to vaccines. Results will display the number of remaining introductions per country in order to identify and focus efforts in areas where there are the most remaining vaccine introductions.  **Frequency of evaluation:** Annual | Global, regional, and country partners can use findings from this indicator to identify countries that are remaining to introduce WHO recommended vaccines in order to focus efforts to ensure equitable access to vaccines. The indicator will help to assess whether there are opportunities to support country vaccination introductions for new and underutilized vaccines.  Further in-depth evaluation or root cause analysis could be considered to determine barriers to vaccine introduction for the countries with remaining introduction of most of the WHO recommended vaccines. |

| **INDICATOR 3.1. SDG 3.b.1 - Coverage of vaccines included in national immunization schedules (DTP3, MCV2, PCV3, and HPVc)** | | |
| --- | --- | --- |
| **MONITOR**  ***How will progress be monitored?*** | **EVALUATE *How will results of monitoring be evaluated?*** | **ACT**  ***How will evaluation be used for action?*** |
| **Definition**: Immunization coverage for DTPcv-3, MCV-2, PCV3 and HPVc  **Measurement approach**: Immunization coverage for a certain year is defined as the proportion of the targeted population that received the relevant vaccine and dose in that year.  Coverage will be determined at country, regional and global levels, using WHO and UNICEF estimates of national immunization coverage (WUENIC). Note that for WUENIC, the annually targeted population for globally recommended vaccines comprises the entire global cohort of surviving infants, regardless of whether the vaccine was introduced in their country.  At the national and subnational level, administrative reporting systems can also be used, together with any in-country survey results and other information sources that can help countries establish coverage estimates.  **Calculation:** Denominator is estimated population of target group of children that should receive DTPcv-3, MCV-2, PCV3 and HPVc. Numerator consists of target population who have received DTPcv-3, MCV-2, PCV3 and HPVc. Target population of children and their appropriate age for last dose is determined by national immunization schedule.  **Data source:** WHO and UNICEF estimates of national immunization coverage (WUENIC)  **Stakeholder(s) responsible for measurement:** WHO IVB, national immunization programmes  **Frequency of reporting**: Annual at regional and global levels, monthly at national and subnational levels. | **Baseline: 85%** DTPcv-3, **71%** MCV-2, **48%** PCV3 and **15%** HPVc (2019)  **Target:**  Global level: **90%** coverage for all by 2030  Country level:   - Plan introduction of all globally recommended vaccines by 2030 - Ensure coverage for each vaccine reaches levels within a 5% range from DTPcv-1   **Analysis and interpretation:** Level and trend, disaggregated by geography and other dimensions (socio-economic, language group, ethnicity) as available.  **Frequency of evaluation:** Annual at global and regional levels. Ideally quarterly at national and subnational levels. | At the global and regional level, coverage estimates will be used for prioritization, and to create accountability for countries that do not reach targets, or backslide from previously attained targets.  Furthermore, coverage estimates can be used to communicate about immunization gaps that exist in the world, and advocate for concerted efforts to bridge them.  At the country and subnational level, measuring the level and trend of coverage, as well as estimates of vaccinated people (numerators), can help establish whether:   - Immunization programmes are showing desired progress overall, by geography, and by population group. - Immunization platforms for the different age groups perform adequately. - Vaccine-specific barriers exist.   Immunization programmes can then implement any corrective action. |

| **INDICATOR 3.2. UHC Index of Service Coverage** | | |
| --- | --- | --- |
| **MONITOR**  ***How will progress be monitored?*** | **EVALUATE *How will results of monitoring be evaluated?*** | **ACT**  ***How will evaluation be used for action?*** |
| **Definition:** The indicator will measure coverage of essential health care services**.**  Coverage of essential health services is defined as the average coverage of essential services based on tracer interventions that include reproductive, maternal, newborn and child health, infectious diseases, non-communicable diseases and service capacity and access, among the general and the most disadvantaged population.  **Measurement approach:**  Indicator SDG 3.8.1 on coverage of essential health services is measured using an index called the UHC Index of service coverage (UHC SCI). The UHC SCI will be the initial metric to measure key aspects of UHC and Primary Health Care (PHC). The metrics used to measure UHC and PHC will likely evolve over the decade with advances in data and understanding. The target for the IG 3.2 indicator will also evolve to ensure immunization programme’s contribution to PHC/UHC is best measured.  **Calculation:** UHC SCI will be reported at the country level. Regional and global means will be calculated.  **Stakeholder(s) responsible for measurement:** WHO IVB; WHO Division of Data, Analytics and Delivery for Impact; Primary Health Care Performance Initiative; WHO Department of Service Delivery and Safety; and UHC2030.  **Data sources:** UHC SCI.  **Frequency of reporting:** Every second year. | **Baseline:** UHC SCI values for 2019  **Target:** Improve UHC Index of Service Coverage at country, regional and global levels over baseline values.  **Analysis and interpretation:**  Indicators of service coverage – defined as people receiving the service they need – are the best way to track progress in providing services under UHC. Since a single health service indicator does not suffice for monitoring UHC, the UHC SCI is constructed from 14 tracer indicators selected based on epidemiological and statistical criteria. The index is reported on a unitless scale of 0 to 100, with 100 being the optimal value.  UHC is defined as ensuring that all people have access to needed health services (including prevention, promotion, treatment, rehabilitation and palliation) of sufficient quality to be effective while also ensuring that the use of these services does not expose the user the financial hardship. The indicator will focus on the service coverage component of the UHC-SCI because immunization programmes’ contribution to UHC is primarily through service coverage.  Progress towards the target will be assessed by monitoring bi-annual trends in the UHC SCI values at the country, regional, and global levels.  **Frequency of evaluation:** Every second year. | Global, regional, and country partners can use evaluation findings for operational planning, and for communication and advocacy to:   - identify potential root causes of success and failure and areas for improvement in increasing the UHC SCI - identify settings with missed opportunities for improved coverage through better integration - ensure needed support to countries to improve UHC SCI as part of health system strengthening efforts - promote alignment of IA2030 and UHC - promote efforts to integrate delivery and utilization of immunization and other UHC/PHC services   At a country and subnational level, monitoring this indicator should particularly help in:   - ensuring immunization programmes are an integral part of national PHC strategies and operations, as well as national strategies for UHC. - strengthen delivery of integrated services as part of PHC, across the life course. - verifying whether health programmes have policies and/or standard operating procedures in place that promote integration between programmes, thereby reducing missed opportunities. |

**Strategic Priority Objective Indicators**

*Additional regional and country indicators for monitoring SP Objectives will be developed by regions and countries for inclusion in their IA2030 M&E plans.*

Table 1: Indicator summary for monitoring SP1 at all levels

| **SP1: IMMUNIZATION PROGRAMMES FOR PHC/UHC** | | |
| --- | --- | --- |
| **SP Objective 1.1: Reinforce and sustain strong leadership, management and coordination of immunization programmes at all levels** | | |
| **Indicator selected for global monitoring** | **Options for regional monitoring** | **Options for country monitoring** |
| Proportion of countries with evidence of adopted mechanism for monitoring, evaluation and action at national and sub-national levels | **Aligns with global-level monitoring:**  Proportion of countries with evidence of adopted mechanism for monitoring, evaluation and action at national and sub-national levels  **Indicator options:**   1. % of countries with district health management committees (or equivalent at subnational level) that review immunization performance as part of primary health care performance at least annually 2. % of countries with up-to-date Immunization Technical Guidelines (not older than 5 years) 3. % of countries with functional Interagency Coordinating Committee (ICC) 4. % of countries with functional National Immunization Technical Advisory Groups (NITAGs) 5. % of countries with functioning Public Health Emergency Operations Centres (PHEOCs), polio or malaria EOCs capable of responding to VPD outbreaks | **Aligns with global-level monitoring:**  Mechanism in place for monitoring, evaluation and action at national and sub-national levels  **Indicator options:**   1. % of district health management committees (or equivalent at subnational level) that review immunization performance as part of primary health care performance at least annually 2. Multisector coordination mechanisms functional at all levels 3. Number of health facilities reached with supportive supervision visit 4. Percentage of facilities that are led by a manager(s) who has official management training (for example, a certification, diploma, or degree) 5. Number of times annually that a Public Health Emergency Operations Centre (PHEOC) or disease-specific EOC is activated for VPD outbreaks |
| **SP Objective 1.2: Ensure the availability of an adequate, effective, sustainable health workforce** | | |
| **Indicator selected for global monitoring** | **Options for regional monitoring** | **Options for country monitoring** |
| Density of physicians, nurses and midwives per 10,000 population | **Aligns with global-level monitoring:**  Density of physicians, nurses and midwives per 10,000 population  **Indicator options:**   - % countries that achieve the recommended density of health workers per 10,000 population (five occupations are monitored within this indicator: medical doctors, nursing personnel, midwifery personnel, dentists, pharmacists) - % of countries with >90% of vaccination posts having trained health staff. - % countries with >25% gap in immunization staff - % of countries with health workforce competencies established | **Aligns with global-level monitoring:**  Density of physicians, nurses and midwives per 10,000 population  **Indicator options:**   1. Health staff competent in immunization per 10,000 population per region 2. Number of health workers per 10,000 population by cadre (nurse, midwife, physician, community health worker) with disaggregation by gender, age, level of service delivery, managing authority, and subnational administrative area. 3. Number and % of service delivery points with a trained vaccinator in the last 2 years 4. Ratio of unfilled posts to total number of posts, by occupation and by subnational level (% vacant positions of nursing and frontline health workers) |
| **SP Objective 1.3: Build and strengthen comprehensive vaccine-preventable disease surveillance as a component of the national public health surveillance system, supported by strong, reliable laboratory networks** | | |
| **Indicator selected for global monitoring** | **Options for regional monitoring** | **Options for country monitoring** |
| Proportion of countries with 90% on-time reporting from 90% of districts for suspected cases of all priority vaccine-preventable diseases included in nationwide surveillance (including reporting of zero cases) | **Aligns with global-level monitoring**:  Proportion of countries with 90% on-time reporting from 90% of districts for suspected cases of all priority vaccine-preventable diseases included in nationwide surveillance (including reporting of zero cases)  **Indicator options:**   - % of countries achieving the non-measles/non-rubella discard rate of ≥2/100,000 persons and the non-polio acute flaccid paralysis rate of >1/100,000 among <15 years population) in a 12-month period - % of countries with access to laboratory capacity to test for at least one bacterial VPD | **Aligns with global-level monitoring:**  % of districts reporting at least 90% on time during a one-year period for suspected cases for all priority VPDs under nationwide surveillance, including reporting of zero cases.  **Indicator options:**   1. Non-polio acute flaccid paralysis rate (target >1/100,000 among <15 years population) in a 12-month period 2. Non-measles/non-rubella discard rate (target ≥2/100,000 population) 3. Access to laboratory capacity to test for at least one bacterial VPD |
| **SP Objective 1.4: Secure high-quality supply chains for vaccines and related commodities and effective vaccine management, within the primary health care supply system** | | |
| **Indicator selected for global monitoring** | **Options for regional monitoring** | **Options for country monitoring** |
| Proportion of time with full availability of DTPcv and MCV at service delivery level (mean across countries) | **Aligns with global-level monitoring:**  Proportion of time with full availability of DTPcv and MCV at service delivery level (mean across countries)  **Indicator options:**   - % of countries having electronic vaccine and supply stock management system to monitor vaccine stock down to service delivery - % countries that carried out Effective Vaccine Management Assessment during the last 3 years - % of countries that achieved > 80% score in at least two of the AQE (A-availability, Q-quality and E-efficiency) EVM indicator categories (EVMA score) - % countries that have regularly updated and complete (min once per 6 months) cold chain inventories (CCI with geolocated CCE) - % of countries below 1% closed vial wastage for PCV | **Aligns with global-level monitoring:**  Full availability of DTPcv and MCV at service delivery level  **Indicator options:**   1. % districts reporting stock availability (vaccines and supplies) at a service delivery level 2. % districts having electronic vaccine and supply stock management system to monitor vaccine stock down to service delivery 3. Stock out events of DTP or MCV at national level 4. Stock out days at national level 5. Stock out events of DTP or MCV at sub-national level 6. Effective Vaccine Management Assessment (EVMA) conducted 7. Percentage of sites with functional PQS equipment 8. EVM score (not just whether it has been conducted) 9. Functionality of cold chain equipment 10. Closed vial wastage for PCV |
| **SP Objective 1.5: Information Systems** | | |
| **Indicator selected for global monitoring** | **Options for regional monitoring** | **Options for country monitoring** |
| None | **Indicator options:**   - Number of countries in the region in which the percentage of population with access to personal immunization records* is ≥80%** * includes both paper-based and digital records.   **targets to be set at regional level.   - Evaluation score (e.g. Countries with Effective Information System Quality ≥90) - % of countries with 90% or more completeness and timeliness reporting - Proportion of countries that have:   - Electronic immunization registers (EIR) with national coverage (i.e. an EIR that covers their entire population of children born in that year)   - An integrated HMIS that includes vaccination data   - A digital health information strategy | **Indicator options:**   1. % of population with access to personal immunization records 2. Availability of sustainable and effective immunization information system integrated within a robust national health information system (HIS) 3. % of districts with on-line access to HMIS 4. % of live births registered 5. Country uses quality data on under-vaccinated to inform plans at community, subnational and national levels 6. % of children with home-based immunization records 7. % of districts with complete and timely reporting 8. Percentage of districts reporting negative DTP1-DTP3 drop out 9. Percent of districts with year-to-year variation of children vaccinated with DTP3 less than 15% |
| **SP Objective 1.6: Vaccine Safety** | | |
| **Indicator selected for global monitoring** | **Options for regional monitoring** | **Options for country monitoring** |
| Proportion of countries with at least one documented (with reporting form and/or line listed) individual serious AEFI case safety report per million total population | **Recommended indicator:**  Proportion of countries that are reporting individual serious AEFI into Vigibase*  **Indicator options:**   1. % of countries where vaccine safety data is shared between the NRA and the immunization programme (i.e. the data on serious AEFI cases reported in the JRF for the previous year is identical to the data uploaded to the Vigibase in the same year based on date of AEFI onset) 2. % of countries with a functional** AEFI committee   *this applies to ALL countries irrespective of access to Vigibase as countries are encouraged to report AEFI cases to Vigibase progressively so that 100% of countries are reporting individual serious AEFI into Vigibase by 2030  ** as described in section 4.6 of the global manual on surveillance of AEFI accessed at <https://www.who.int/vaccine_safety/publications/Global_Manual_revised_12102015.pdf?ua=1> | **Aligns with global-level monitoring:**  Individual AEFI case safety reports per million total population  **Indicator options:**   1. Proportion of provinces/districts or other subnational units with at least one documented (with reporting form and/or line listed) individual serious AEFI case safety reports per million total population 2. Proportion of serious* AEFI cases where causality assessment was done   *An event that results in death, is life-threatening, requires in-patient hospitalization or prolongation of existing hospitalization, results in persistent or significant disability/ incapacity, or is a congenital anomaly/birth defect. Any medical event that requires intervention to prevent one of the outcomes above may also be considered as serious. |

Table 2: Indicator summary for monitoring SP2 at all levels

| **SP2: COMMITMENT & DEMAND** | | |
| --- | --- | --- |
| **SP Objective 2.1: Build and sustain strong political commitment for immunization at all levels** | | |
| **Indicator selected for global monitoring** | **Options for regional monitoring** | **Options for country monitoring** |
| Proportion of countries with legislation in place that is supportive of immunization as a public good | **Aligns with global-level monitoring:**  Proportion of countries with legislation in place that is supportive of immunization as a public good | **Aligns with global-level monitoring:**  Legislation is in place that is supportive of immunization as a public good  **Indicator options:**   - Commitment tracking and accountability frameworks used at country and subnational levels |
| **SP Objective 2.2: Ensure that all people and communities value, actively support and seek out immunization services** | | |
| **Indicator selected for global monitoring** | **Options for regional monitoring** | **Options for country monitoring** |
| Proportion of countries that have implemented behavioural or social strategies (i.e. demand generation strategies) to address under-vaccination | **Aligns with global-level monitoring:**  Proportion of countries that have implemented behavioural or social strategies (i.e. demand generation strategies) to address under-vaccination  **Indicator options:**   1. Government support for community action (e.g. earmarked funds for community action, provision of technical tools tailored to communities, programmes for subgroups at particular risk) 2. Countries with dedicated online resource for sharing accurate information about vaccines and immunization, including local schedule 3. Countries with routine digital listening platforms established | **Aligns with global-level monitoring:**  Implementation of behavioural or social strategies (i.e. demand generation strategies) to address under-vaccination in the previous year  **Indicator options:**   1. Health facility microplans that include engagement with civil society and community representatives 2. Health facilities with staff that received training (refresher or other) on interpersonal communications or similar 3. % of population that values vaccination 4. Placeholder for additional BeSD-based indicator 5. Placeholder for programmatic indicator on overcoming gender-related barriers to immunization |

Table 3: Indicator summary for monitoring SP3 at all levels

| **SP3: COVERAGE & EQUITY** | | |
| --- | --- | --- |
| **SP Objective 3.1: Extend immunization services to regularly reach “zero-dose” and under-immunized children and communities** | | |
| **Indicator selected for global monitoring** | **Options for regional monitoring** | **Options for country monitoring** |
| None | **Indicator options:**   1. Number of countries with evidence-based and funded plan to address coverage of high-risk communities (zero-dose and under-immunized) 2. % of countries with strategies to reach disadvantaged population 3. % of countries that include activities to reach zero-dose children and missed communities in their national immunization strategies 4. % of countries for which at least 80%* of districts have microplans that specifically target zero-dose communities (*target to be set at regional level) 5. % of countries that have conducted an analytic assessment (coverage and equity analysis) of the number and distribution of zero-dose and underimmunized children and the determinants of missed communities 6. Number of immunization sessions conducted, disaggregated by delivery type. | **Indicator options:**   1. Evidence-based and funded plan to address coverage of high-risk communities exists 2. Dropout rates between first dose (DTP1) and third dose (DTP3) of DTP-containing vaccines; and dropout rates between DTP1 and MCV1 3. Number of immunization sessions (1) planned and (2) conducted, disaggregated by delivery type (e.g. fixed, outreach). 4. % of districts in which at least 80% of planned (outreach) sessions are also held 5. % of eligible children in the disadvantaged population that are reached and vaccinated according to national schedule. 6. % of districts with (micro) plans that specifically target zero-dose and under-immunized communities 7. % of acute flaccid paralysis (AFP) cases who are identified as being “zero dose” or previously unvaccinated with OPV |
| **SP Objective 3.2: Advance and sustain high and equitable immunization coverage nationally and in all districts** | | |
| **Indicator selected for global monitoring** | **Options for regional monitoring** | **Options for country monitoring** |
| DTP3, MCV1, and MCV2 coverage in the 20% of districts with lowest coverage (mean across countries) | **Aligns with global-level monitoring:**  DTP3, MCV1, and MCV2 coverage in the 20% of districts with lowest coverage (mean across countries)    **Indicator options:**   1. % of countries with annualized national dropout rate of DTPcv1 and DTPcv3 greater than 5% points 2. % of countries that have explicit strategies in their national immunization strategies to overcome gender-related barriers to vaccination | **Aligns with global-level monitoring:**  DTP3, MCV1, and MCV2 coverage in the 20% of districts with lowest coverage  **Indicator options:**   1. Dropout rates between first dose (DTP1) and third dose (DPT3) of DTP-containing vaccine 2. Geographic equity of immunization coverage 3. Percentage points difference in coverage of DTPcv1, MCV1 and FIC associated with the most important socio-economic determinants of vaccination coverage in the country (poverty, education, ethnicity, religious affiliation) 4. % of population living within 5 km to a fixed-site facility offering immunization services |

Table 4: Indicator summary for monitoring SP4 at all levels

| **SP4: LIFE COURSE & INTEGRATION** | | |
| --- | --- | --- |
| **SP Objective 4.1: Strengthen immunization policies and service delivery throughout the life course, including for appropriate catch-up vaccination and booster doses** | | |
| **Indicator selected for global monitoring** | **Options for regional monitoring** | **Options for country monitoring** |
| Breadth of protection (mean coverage for all WHO-recommended vaccine antigens, by country) | **Aligns with global-level monitoring:**  Breadth of protection (mean coverage for all WHO-recommended vaccine antigens, by country)    **Indicator options:**   1. Proportion of countries with at least three vaccines targeting population beyond the first year of life in the national immunization schedule 2. Proportion of countries with MCV2, DTP-containing vaccine (DTPcv) booster dose, HPV in the national immunization schedule. 3. Proportion of countries with seasonal influenza vaccination programmes for either all individuals or targeted high-risk sub-populations | **Aligns with global-level monitoring:**   - Proportion of WHO recommended vaccines present within their national immunization schedule**.**   **Indicator options:**   1. Number of vaccines targeting population beyond the first year of life in the national immunization schedule 2. Availability of policies and/or laws for vaccination in childhood, adolescence and adulthood, including policies on catch-up of earlier missed vaccinations 3. Coverage of MCV2, Penta booster dose, HPV 4. Coverage of seasonal influenza vaccination in countries that include it in the national immunization schedule for all individuals or targeted high-risk sub-populations 5. Percentage of LQAs achieving >80% “pass” rate during SIA campaigns, such as polio, measles, etc. |
| **SP Objective 4.2: Establish integrated delivery points of contact between immunization and other public health interventions for different target age groups.** | | |
| **Indicator selected for global monitoring** | **Options for regional monitoring** | **Options for country monitoring** |
| None | **Indicator options:**   1. Proportion of countries with national policies or standard operating procedures in place to strengthen delivery of immunization services integrated with primary health care, across the life course 2. Proportion of countries with national guides for service delivery integration to prevent missed opportunities, for all age groups 3. Proportion of countries with >90% of PHC providing immunization services. 4. Proportion of countries with >80% of tertiary health care providing immunization services 5. Proportion of countries integrating immunization delivery in ≥90% of existing non-traditional delivery strategies (e.g. schools, pharmacies) 6. Proportion of countries with a composite coverage index (CCI) (e.g. GVAP integration indicator G5.2) stratified by CCI < 60 (weak health systems), CCI 60–70 (less weak health systems), CCI > 70 (stronger health systems) 7. Proportion of countries that link home-based records (HBR) with civil birth registration through immunization services | **Indicator options:**   1. National policies or standard operating procedures in place to strengthen delivery of immunization services integrated with primary health care, across the life course 2. Existence of national guides for service delivery integration to avoid missed opportunity, for all age groups 3. % of existing non-traditional delivery strategies (e.g. schools, pharmacies) integrating immunization delivery 4. % of PHC centres integrating immunization services with other PHC services 5. % of tertiary health care providing daily immunization service 6. Linkage of home-based records (HBR) with civil birth registration through immunization services 7. % of immunization clinics with an active mechanism to offer post-partum family planning in the first year after childbirth 8. Number of districts, and % coverage, with routine well child checks in second year of life that include growth, nutrition and vaccination 9. Number of districts with active investigation of the % of missed opportunities for vaccination (MOV) using the WHO MOV strategy in annual immunization plans |
| **SP Objective 4.3: Accelerate new vaccine introductions to protect more people from more diseases in all countries** | | |
| **Indicator selected for global monitoring** | **Options for regional monitoring** | **Options for country monitoring** |
| None | **Indicator options:**   1. Proportion of countries with all WHO-recommended vaccines within their national immunization schedule 2. Proportion of countries with newly recommended vaccines introduced post-2020. | **Indicator options:**   1. Proportion of all WHO-recommended vaccines within their national immunization schedule within X years of WHO policy recommendation. 2. Proportion of each life course stage reached with the last dose of WHO-recommended vaccines 3. % of coverage of newly recommended vaccines introduced post-2020. 4. Rate of scale up of new vaccines 5. Number of vaccine introductions |

Table 5: Indicator summary for monitoring SP5 at all levels

| **SP5: OUTBREAKS & EMERGENCIES** | | |
| --- | --- | --- |
| **SP Objective 5.1: Ensure preparation for, detection of, and rapid, high-quality responses to vaccine-preventable disease outbreaks** | | |
| **Indicator selected for global monitoring** | **Options for regional monitoring** | **Options for country monitoring** |
| Proportion of polio, measles, meningococcal disease, yellow fever, cholera, and Ebola outbreaks with timely detection and response (includes only outbreaks with an outbreak response vaccination campaign) | **Aligns with global-level monitoring:**  Proportion of polio, measles, meningococcal disease, yellow fever, cholera, and Ebola outbreaks with timely detection and response (includes only outbreaks with an outbreak response vaccination campaign)  **Indicator options:**   1. Annual number of laboratory-confirmed epidemic-prone vaccine-preventable disease outbreaks 2. For epidemic-prone vaccine-preventable diseases, average coverage achieved by outbreak response vaccination campaigns 3. % of countries with national outbreak response plan | **Aligns with global-level monitoring**:  Proportion of polio, measles, meningococcal disease, yellow fever, cholera, and Ebola outbreaks with timely detection and response  **Indicator options:**   1. Annual number of laboratory-confirmed epidemic-prone vaccine-preventable disease outbreaks 2. For epidemic-prone vaccine-preventable diseases, average coverage achieved by outbreak response vaccination campaigns 3. (National outbreak response plan developed - Y/N) 4. % of stockpile applications that demonstrate use of evidence (e.g. disease surveillance data, root cause analysis, and coverage data) to support planning/targeting of outbreak response campaigns |
| **SP Objective 5.2: Establish timely and appropriate immunization services during emergencies, and in communities affected by conflict, disaster and humanitarian crisis** | | |
| **Indicator selected for global monitoring** | **Options for regional monitoring** | **Options for country monitoring** |
| None | **Indicator options:**  Percentage of children who have age-appropriate vaccination for DTP3, MCV (last dose), and PCV (last dose) in settings with humanitarian crises or emergencies | **Indicator options:**   1. Annual % of children who have age-appropriate vaccination coverage for DTP3, MCV (last dose), and PCV (last dose) in settings with humanitarian crises or emergencies 2. Number of zero-dose and underimmunized children in fragile, conflict and emergency settings 3. SMART or equivalent vaccine surveys carried out during a year of crisis |

Table 6: Indicator summary for monitoring SP6 at all levels

| **SP6: SUPPLY & SUSTAINABILITY** | | |
| --- | --- | --- |
| **SP Objective 6.1: Build and maintain healthy global markets across all vaccine antigens** | | |
| **Indicator selected for global monitoring** | **Options for regional monitoring** | **Options for country monitoring** |
| Level of health of the vaccine market, disaggregated by vaccine antigens and country typology | **None** | **None** |
| **SP Objective 6.2: Ensure sufficient financial resources for immunization programmes in all countries** | | |
| **Indicator selected for global monitoring** | **Options for regional monitoring** | **Options for country monitoring** |
| Proportion of countries whose domestic government and donor expenditure on primary health care increased or remained stable, constant prices per capita (GGHE indicator) | **Aligns with global-level monitoring:**  Proportion of countries whose domestic government and donor expenditure on primary health care increased or remained stable  **Indicator options:**   1. Number of countries with stagnant or increased government expenditure (broken down by domestic and donor funding) – in constant prices per live birth – on immunization (breaking down vaccine and estimated operational cost) 2. Number of countries where immunization was de-prioritized, i.e.  - share of domestic public budget allocated to immunization (vaccines and operational cost) declined - share of public budget – including donor money – allocated to immunization declined  1. Number of countries that track immunization expenditure using health accounts | **Aligns with global-level monitoring:**  Increasing or stagnant trend of domestic government and donor expenditure on primary health care and on immunization, in constant prices per capita and live birth  **Indicator options:**   1. Has the share of domestic government only on primary health care and on immunization, in constant Ps per capita and live birth, increased? 2. Is the annual execution rate of immunization budget less than 90%? 3. Is the annual execution rate of PHC budget less than 90%? 4. Is there an annual operational plan in place, stipulating the needs for the programme and the available resources to cover those needs? 5. Has an analysis for the financing of immunization been conducted recently to identify bottlenecks to progress towards universal access to immunization, and explore possibilities for efficiency gain through integrated services? |
| **SP Objective 6.3: Increase immunization expenditure from domestic resources in aid-dependent countries, and when transitioning away from aid, secure government funding to achieve and sustain high coverage for all vaccines** | | |
| **Indicator selected for global monitoring** | **Options for regional monitoring** | **Options for country monitoring** |
| Proportion of countries whose share of national immunization schedule vaccine expenditure funded by domestic government resources increased | **Aligns with global-level monitoring:**  Proportion of countries whose share of national immunization schedule vaccine expenditure funded by domestic government resources increased  **Indicator options:**  Number of countries with stagnant or increased share of immunization schedule vaccine expenditure funded by domestic government resources | **Aligns with global-level monitoring:**  Percentage of total expenditure on vaccines in the national immunization schedule financed with domestic government funds  **Indicator options:**  The cost reduction of vaccines over time, by antigen |

Table 7: Indicator summary for monitoring SP7 at all levels

| **SP7: RESEARCH & INNOVATION** | | |
| --- | --- | --- |
| **SP Objective 7.1: Establish and strengthen capacity at all levels to identify priorities for innovation, and to create and manage innovation** | | |
| **Indicator selected for global monitoring** | **Options for regional monitoring** | **Options for country monitoring** |
| Proportion of countries with an immunization research agenda | **Aligns with global-level monitoring:**  Proportion of countries with an immunization research agenda  **Indicator options:**   1. Availability of common framework/process/format for developing country immunization research agendas 2. Proportion of countries that developed immunization research agenda, relative to baseline 3. Proportion of countries that have secured funding to develop and implement national immunization agendas 4. Proportion of countries engaged in vaccine product and delivery, R&D, implementation research and/or manufacturing | **Aligns with global-level monitoring:**   - Immunization research agenda exists   **Indicator options:**   1. Improved institutional and technical capacity to carry out vaccine clinical trials 2. Number of districts that have identified their priorities for new products/innovations |
| **SP Objective 7.2: Develop new vaccines and technologies, and improve existing products and services for immunization programmes** | | |
| **Indicator selected for global monitoring** | **Options for regional monitoring** | **Options for country monitoring** |
| Progress towards global research and development | **Indicator options:**   1. Number of new vaccine-related products/innovations approved/implemented or in pilot studies 2. Number of pivotal clinical trials performed 3. Number new vaccines prequalified 4. Number of countries with RITAG approving their research immunization agenda priorities 5. Number of vaccines in commercial manufacture 6. The establishment of an evaluation framework to assess uptake and implementation effectiveness of new and existing products and services | **Indicator options:**   1. Number of new vaccine-related products/innovations approved by national regulatory authority (NRA) 2. Number of new vaccines recommended for use 3. Number of pivotal clinical trials performed 4. Number of vaccines in commercial manufacture |
| **SP Objective 7.3: Evaluate promising innovations and scale up innovations as appropriate based on the basis of the best available evidence** | | |
| **Indicator selected for global monitoring** | **Options for regional monitoring** | **Options for country monitoring** |
| None | **Indicator options:**   1. Proportion of countries that have established processes/frameworks for identifying vaccine products and innovations to develop, introduce or use 2. Proportion of countries with at least one implemented recommendation from a NITAG or other relevant independent technical advisory group | **Indicator options:**   1. At least one implemented recommendation from a NITAG or other relevant independent technical advisory group implemented 2. List of evidence-based solutions to strengthen immunization service delivery 3. Progress implementing/scaling up evidence-based solutions to strengthen immunization service delivery |

**Metadata for Strategic Priority Objective Indicators**

**Global Strategic Priority Objective Indicators**

*Each Global SP Objective Indicator will be defined with the following characteristics*:

| **Indicator ID, Name** | **SP 1.1- Proportion of countries with evidence of adopted mechanism for monitoring, evaluation and action at national and sub-national levels** |
| --- | --- |
| Definition | **Mechanism that drives monitoring, evaluation and action (ME&A) cycles at national and sub-national (equivalent to district) levels is defined according to the following criteria.**  **Criteria include:**   1. Presence of a functional NITAG or equivalent technical advisory group 2. Monitoring, evaluation and action cycles are in place 3. Feedback loop is in place to communicate assessments of progress, and recommendation actions from sub-national to national and from national to sub-national level |
| Calculation and operational considerations | Data-driven decision-making is an indication of strong leadership and management. This indicator should help in uniting the key stakeholders to drive actions in an accountable manner. Information from ME&A exercises should be reported to higher levels, and recommendations fed back to lower levels. Actions planned/taken should be reported to higher levels and from higher to lower levels.  The indicator will be self-reported according to the criteria above. Meeting each criterion gives 1 point, with a maximum score of 3 points. Data for this indicator is not currently available at the global level, except for NITAG presence and functionality which is collected through the JRF.  **JRF questions:**  **Criteria 1**: Presence of a functional NITAG or equivalent technical advisory group (already collected through JRF).  “Functional” defined as meeting the following: *1. Technical advisory group has a formal written terms of reference; 2. There is a legislative or administrative basis for the advisory group; 3. The following areas of expertise are represented in the group as core membership: pediatrics; public health; infectious diseases; epidemiology; immunology; 4. members of the technical advisory group are required to disclose conflict of interest; 5. Committee meets at least once a year on a regular basis; and 6. agenda and background documents distributed to technical advisory group members at least 1 week ahead of meetings.*  **Criteria 2**:  Monitoring, evaluation and action cycles were in place in [insert previous year].   1. In [insert previous year], did your country have monitoring, evaluation, and action (ME&A) cycles in place for data-driven decision making? 2. If yes, please share an operational document describing the ME&A process in your country in the previous year: 3. If yes, please provide a description summary of implemented actions to strengthen immunization programme performance that occurred through the implementation of monitoring, evaluation, and action cycles. 4. If yes, select stakeholders that provided guidance for monitoring, evaluation, and action cycles (ME&A) in [insert previous year]    1. NITAG    2. Government    3. CSOs    4. Other (specify)   **Criteria 3:** Feedback loop is in place to communicate assessments of progress, and recommendation actions from sub-national to national and from national to sub-national level  3.1 In [insert previous year], was the evaluation of immunization indicator results communicated from national to subnational levels?  3.2 In [insert previous year], was the evaluation of immunization indicator results communicated from subnational levels to the national level? |
| Method of measurement | Data for this indicator will be collected through self-report (Yes/No) and a request to provide supporting documentation.  Supporting documentation will include:   - Operational document describing the ME&A process at all levels - Evidence of implemented actions to strengthen immunization programme performance at all levels |
| Data source | Proposed to be collected through JRF |

| **Indicator ID, Name** | **SP 1.2- Density of physicians, nurses and midwives per 10,000 population** |
| --- | --- |
| Definition | Number of physicians, nurses and midwives per 10,000 population |
| Calculation and operational considerations | Density of physicians, nurses and midwives per 10,000 population:  Numerator: Number of physicians, nurses and midwives, defined in headcounts  Denominator: Total population (per 10,000)  Physicians comprise the following occupations: generalists, specialist medical practitioners and medical doctors. The International Standard Classification of Occupations (ISCO) unit group codes included in this category are 221, 2211, 2212.  Nursing and midwifery personnel comprise the following occupations: nursing professionals, nursing associate professionals, midwifery professionals, midwifery associate professionals and related occupations. The ISCO unit group codes included in this category are 2221, 2222, 3221 and 3222 of ISCO-08. |
| Method of measurement | In response to WHA resolution, WHA 69.19, an online National Health Workforce Accounts (NHWA) data platform was developed to facilitate reporting. Complementing national reporting through the NHWA data platform, additional sources such as the National Census, Labour Force Surveys and key administrative national and regional sources are also employed. In general, the denominator data for workforce density (i.e. national population estimates) are obtained from the United Nations Population Division's World Population Prospects database. In cases where the official health workforce report provides density indicators instead of counts, estimates of the stock were then calculated using the population estimated from the United Nations Population Division's World population prospects database.  Further information:  <https://www.who.int/data/gho/data/indicators/indicator-details/GHO/nursing-and-midwifery-personnel-(per-10-000-population)>  <https://www.who.int/activities/improving-health-workforce-data-and-evidence> |
| Data source | Numerator: WHO National Health Workforce Accounts (NHWA)  Denominator: UN Population Division’s World Population Prospects |

| **Indicator ID, Name** | **SP 1.3- Proportion of countries with 90% on-time reporting from 90% of districts for suspected cases of all priority vaccine-preventable diseases included in nationwide surveillance (including reporting of zero cases)*** |
| --- | --- |
| Definition | Countries with on time reporting from districts of suspected cases of all priority VPDs included in nationwide surveillance (including reporting of zero cases)  *suspected cases for all priority VPDs under nationwide surveillance. Priority VPDs include at a minimum, polio, measles, rubella, neonatal tetanus, yellow fever (for endemic countries), meningococcal (for meningitis belt countries) and other diseases under nationwide surveillance that a country/region determines is a priority. |
| Calculation and operational considerations | Denominator - total countries reporting data.  Numerator consists of countries where at least 90% of districts have achieved at least 90% on time reporting for all priority vaccine-preventable diseases included in nationwide surveillance (including reporting of zero cases)  -   Report the number of cases for all suspected cases of the predefined VPDs to the provincial or national level. The number of cases can be zero  -   Submit those reports in a timely manner as defined by the country’s internal deadlines for reporting.  -   To achieve 90% reporting per year: If a country expects weekly reporting for a given disease then the district needs to report ≥47 times by the deadline set by the country. If they have monthly reporting for a disease, then reporting should be ≥11 times in a calendar year.  Districts will not count in the numerator if they:  -   Report the number of suspected cases for some, but not all, of the predefined VPDs  -   Do not report on time  -   Report less than 90% of the time.  Countries that are small can use their primary administrative unit or health facilities as their unit of measure |
| Method of measurement | To calculate this indicator, the following questions are proposed for the JRF.   1. What are the priority VPDs in your country (priority VPDs are those that a country defines as those that they want to achieve high quality surveillance to drive their vaccination programme. These could be diseases targeted for elimination/eradication or those that a country is looking at for vaccine introduction or because disease burden is high or because they are highly outbreak prone) that are included in nationwide surveillance (drop down/select all of all VPDs) 2. Number of districts: ____ 3. Please fill in the table to help calculate the indicator.  \| Priority VPD (select all priority VPDs) \| Frequency of reporting (daily, weekly, monthly) \| Total number of districts that reported at any time during the ____ (year) \| Number of districts that report at least 90% on the time during the ___ (year) \| \| --- \| --- \| --- \| --- \| \| Fill in name of VPD 1 \|  \|  \|  \| \| Fill in name of VPD 2 \|  \|  \|  \| \| Fill in name of VPD 3 (etc. through all VPD) \|  \|  \|  \| |
| Data source | Proposed to be collected through JRF |

| **Indicator ID, Name** | **SP 1.4- Proportion of time with full availability at service delivery level of DTPcv and MCV (mean across countries)** |
| --- | --- |
| Definition | Average over all reporting countries of the percentage of health facilities that reported no stock-outs for the full year for DTPcv and MCV |
| Calculation and operational considerations | Countries that report having a system in place to measure vaccine availability at the service delivery level will consolidate facility-level data and calculate the yearly average % of facilities with no stock-outs of DTPcv and MCV.  The following questions are proposed to be added to the JRF:   - Does your country have a system in place to measure vaccine availability at the service delivery level (Y/N)? - What was the availability of measles-containing vaccine in [insert previous year] – defined as the percentage of health facilities that reported no stock outs for the full year? - What was the availability of DTP containing vaccine in [insert previous year]  – defined as the percentage of health facilities that reported no stock-outs for the full year?   Calculation:  Average percentage of DTPcv and MCV across all countries that have a system in place to collect this information.   Operational considerations:  In the context of this indicator, this means for each month, every health facility was able to meet all vaccine needs and reported no stock-outs for the full year for both vaccines. |
| Method of measurement | Countries to monitor and collect facility-level data on DTP containing vaccine and MCV full stock availability over a year using existing information system (e.g. LMIS, HMIS, DHIS2, wVSSM or other available information management platforms).  Countries that lack this data (e.g. no reporting of the indicator, no system to keep track of stock at service delivery level) indicate N for the first question. |
| Data source | Proposed to be collected through JRF |

| **Indicator ID, Name** | **SP 1.6- Proportion of countries with at least 1 documented (with reporting form and/or linelisted) individual serious AEFI* case safety report per million total population** |
| --- | --- |
| Definition | Countries with documented (with reporting form and/ or line-listed) individual serious AEFI case safety reports per million total population |
| Calculation and operational considerations | Annual number of individual AEFI case safety reports available in the WHO global database for safety monitoring.  Threshold: All countries with at least 1 AEFI individual case safety report/1, 000 000 population.  Total population: UN Population Division’s World Population Prospects for e.g.<https://population.un.org/wpp/Publications/Files/WPP2019_Highlights.pdf>  * WHO global database – VigiBase:<https://www.who-umc.org/vigibase/vigibase/> |
| Method of measurement | Individual serious AEFI reporting rate in million total population per year= Number of individually documented serious AEFI cases reported from country/sub-national area per year / Total population in the same country/sub-national area per year * 1,000,000  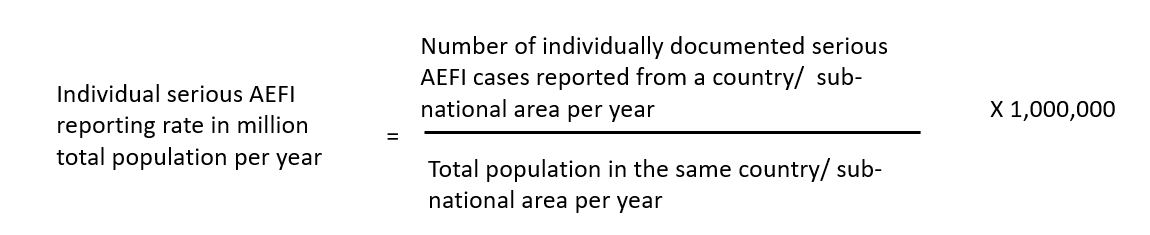 |
| Data source | Primary data source:  WHO global database VigiBase:<https://www.who-umc.org/vigibase/vigibase/> .  VigiBase data will be used for countries which have capacity to upload data to VigiBase.  JRF will be used temporarily for countries that are transitioning to case based reporting into VigiBase. |

| **Indicator ID, Name** | **2.1 Proportion of countries with legislation in place that is supportive of immunization as a public good** |
| --- | --- |
| Definition | Proportion of countries with legislation in place that is supportive of immunization as a public good |
| Calculation and operational considerations | This data is currently not systematically collected at the global level so will need to be added to the JRF in 2021. Calculation will be through self-report (Yes/No) by countries and request to upload a copy or link to the relevant legislation.  Proposed JRF Questions:   - Do you have a vaccination law or other legislation that is supportive of immunization and commits the government to finance all aspects of the immunization programme at all levels?   - Please provide the year it was passed  - Please upload supporting document or provide the website link in the comment field: |
| Method of measurement | The existence (or not) of a legislative basis underlying the commitment to provide government-funded immunization to the population. This will be measured through self-report (Yes/No) and a request to provide supporting documentation. |
| Data source | To be included in JRF. Note: PAHO is piloting this question on the 2020 JRF. Based on feedback from the countries in the region, the exact phrasing of the question may be modified accordingly.  Question will also contain an explanatory note (sample text below):  A “vaccination law or other legislation” could include written laws (acts, statutes) or regulations, orders or decrees established by public authority and enforceable by law. Legislation may be specialized for immunization or be contained in other general public health legislation and, among other things, must consider securing financing for all components of the Immunization Programme at all levels, including the purchase and timely availability of vaccines in accordance with national planning, training, supervision, outreach activities, information systems, and others. |

| **Indicator ID, Name** | **2.2 Percentage of countries that have implemented behavioral or social strategies (i.e. demand generation strategies) to address under-vaccination** |
| --- | --- |
| Definition | Percentage of countries that have implemented behavioral or social strategies (i.e. demand generation strategies) to address under-vaccination |
| Calculation and operational considerations | This data is currently not collected at the global level so will need to be added to the JRF in 2021. Calculation will be through self-report by countries to the following question:  In [insert previous year] did the country implement any behavioral or social strategies (i.e., demand generation strategies) to address under-vaccination? Choose all that apply:   - Interventions to improve access to vaccination - Interventions to improve service quality - Interventions to build capacity among healthcare workers - Community engagement - Interventions to communicate or educate the public - Interventions to manage misinformation based on social or digital listening data - Interventions at the policy level (e.g. incentives) - Other, please specify: |
| Method of measurement | Indicator to be reported by countries through the JRF and will replace former demand questions in the JRF |
| Data source | Proposed to be collected through JRF |

| **Indicator ID, Name** | **3.2: DTP3, MCV1, and MCV2 coverage in 20% of districts with the lowest coverage (mean across countries)** |
| --- | --- |
| Definition | Average over all reporting countries of coverage for DTP3, MCV1, and MCV2 in each country’s 20% lowest-performing districts |
| Calculation and operational considerations | Average coverage in the lowest-performing quintile for each country that reports district level coverage.  Group of worst performing districts may change from year to year (i.e. no attempt to follow the performance in a fixed group of districts) |
| Method of measurement | Analysis of district-level coverage reported by member states. |
| Data source | Annual member state reporting of district-level coverage data through the Joint Reporting Form process |

| **Indicator ID, Name** | **4.1 Breadth of protection: mean coverage for all vaccine antigens recommended by WHO** |
| --- | --- |
| Definition | Breadth of protection defined as mean coverage for all vaccine antigens recommended by WHO |
| Calculation and operational considerations | The average of the coverage achieved at global, regional, country level for the following antigens:   - Diphteria, Tetanus, Pertussis, Hepatitis B, Hib, Measles, Measles 2nd dose, Pneumo, Polio, IPV, Rubella, Rota, HPV   Note that this definition may be further refined. |
| Method of measurement | Analysis of WUENIC |
| Data source | JRF, WUENIC |

| **Indicator ID, Name** | **5.1 Proportion of polio, measles, meningococcal disease, yellow fever, cholera and Ebola outbreaks with timely detection and response (includes only outbreaks with an outbreak response vaccination campaign)** |
| --- | --- |
| Definition | Proportion of polio, measles, meningococcal disease, yellow fever, cholera, and Ebola outbreaks* with timely** detection and response  *Only applies to outbreaks for which there is an outbreak response vaccination campaign.  **Acceptable time from onset of outbreak to campaign implementation to be defined for each disease |
| Calculation and operational considerations | Time from onset of outbreak to implementation of vaccination campaign should be determined for each polio, measles, meningococcal disease, yellow fever, cholera, and Ebola outbreak for which there is an outbreak response vaccination campaign.  Maximum time for the period from onset of outbreak to implementation of vaccination campaign to be considered timely will be defined for each vaccine. Criteria for determining onset of outbreak and timeliness of outbreak detection and response to be consistent with WHO surveillance standards and disease eradication, elimination, or control strategies.  Calculation of indicator will involve division of collective total number of known polio, measles, meningococcal disease, yellow fever, cholera, and Ebola outbreaks with timely detection and outbreak response vaccination campaigns by collective total number of known polio, measles, meningococcal disease, yellow fever, cholera, and Ebola outbreaks with outbreak response vaccination campaigns. |
| Method of measurement | Information from the International Coordinating Group on vaccine provision, Measles Rubella Initiative, Global Polio Eradication Initiative, and WHO World Health Emergencies group, supplemented by national immunization and disease surveillance programs via the WHO/UNICEF Joint Reporting Form. |
| Data source | ICG, MRI, GPEI, WHO, national immunization and disease surveillance programs.  Information will be systematically collected from national immunization and disease surveillance programs to provide data for regional and global level data. |

| **Indicator ID, Name** | **6.1 Level of health of the vaccine market, disaggregated by antigen and country typology** |
| --- | --- |
| Definition | Level of health of the market, disaggregated by antigen and country typology (Gavi, non-Gavi MICs, HICs) |
| Calculation and operational considerations | - Global supply exceeds global demand by more than x and by no more than y – x and y as defined in the MI4A vaccine-specific market studies: <https://www.who.int/immunization/programmes_systems/procurement/mi4a/platform/module2/en/>, by antigen - The 2 largest suppliers do not exceed 2/3 of the market, by antigen - Total number of manufacturers exceeds 3 including the ones with product in clinical development (at least phase IIa), by antigen |
| Method of measurement | A number of criteria have been defined to determine the level of health of a market. The number of criteria ‘met’ directly determine the health of the market for each vaccine. Each organization provides their inputs, and an adjustment exercise is undertaken in case of misalignment.  More specifically the following attribute will be measured:   - supply meeting demand - individual supplier risk - buffer capacity - long term competition   Semi-quantitative assessment of the individual market health will be conducted by partners [WHO, UNICEF, Gavi, BMGF]. Based on assessments of individual antigen the above attributes and a holistic overview of each market’s programmatic context, markets will be assessed based on the following categories:   - Insufficient and requires further intervention: severe supply security challenges and risks exist, no improvement is expected without Gavi Alliance intervention - Insufficient with conditions for improvement: severe supply security challenges and risks exist, improvements possible but requiring further monitoring and lead time to materialize. - Sufficient with risks: limited supply security challenges with unacceptable risks of backsliding, interventions are required to mitigate risks. - Sufficient and sustainable: limited supply security challenges with acceptable risks, monitoring required to ensure risks to not increase. |
| Data source | 1. UNICEF 2. WHO: via the MI4A initiative 3. Gavi Secretariat 4. BMGF |

| **Indicator ID, Name** | **6.2 Proportion of countries whose domestic government and donor expenditure on primary health care increased or remained stable** |
| --- | --- |
| Definition | Proportion of countries whose current government expenditure level (from domestic and donor funding) on primary health care (PHC) per capita in US$ (constant prices) increased or remained stable since pre-2020 level. |
| Calculation and operational considerations | Per capita constant US$ PHC expenditure data is calculated using PHC expenditure, divided by population and measured in constant US$ price (converted in 2020 NCU price and then converted into 2020 US$).  The trend calculation will be defined subsequently, leveraging methodologies used for WHO GHED and GHER (Global Health Expenditure Report). |
| Method of measurement | To monitor growth, proposed methodology is to take the annual growth rates, using constant prices per capita values.  See <https://apps.who.int/nha/database/DocumentationCentre/GetFile/57752201/en> |
| Data source | WHO GHED (health accounts data)  <https://apps.who.int/nha/database/Select/Indicators/en> |

| **Indicator ID, Name** | **6.3 Proportion of countries whose share of national immunization schedule vaccine expenditure funded by domestic government resources increased** |
| --- | --- |
| Definition | Number of countries whose share of current expenditure on vaccines (in the national immunization schedule) that is financed with domestic government funds increased since pre-2020 level. |
| Calculation and operational considerations | The share is calculated from domestic government spending on vaccine as a % of total expenditure on routine immunization vaccines.  The trend calculation will be defined subsequently, leveraging methodologies used for WHO GHED and GHER (Global Health Expenditure Report). |
| Method of measurement | To monitor growth, proposed methodology is to compare shares of Yt with Yt-1.  The total value of vaccines used for the provision of immunization. All the materials and services are to be fully consumed during the production activity period.  Domestic public resources spent on all vaccines used in conformity with the national immunization programme, including routine doses of vaccines, and following each country’s vaccination schedule. Includes the international market price, as well as transport and handling expenditures. Vaccines used in Child Health Days are included in routine vaccine expenditures, but expenditures related to doses of vaccine given through supplemental immunization activities (SIAs) are excluded |
| Data source | JRF |

| **Indicator ID, Name** | **SP 7.1- Proportion of countries with national agenda for research on immunization** |
| --- | --- |
| Definition | Number of countries with national agenda for research on immunization defined and based on clearly identified and prioritized evidence needs and specified in national immunization strategy or other national strategy document |
| Calculation and operational considerations | The national agenda should identify priority research areas that increase the likelihood that the country will achieve its IA2030 targets.  Research is defined as activities that span 5 areas:   - measuring the magnitude and distribution of a health problem; - understanding the diverse causes or the determinants of the problem, whether they are due to biological, behavioral, social or environmental factors; - identifying and developing solutions or interventions that will help to prevent or mitigate the problem; - implementing or delivering solutions through policies and programmes; and - evaluating the impact of these solutions on the magnitude, level and distribution of the problem.   Research agendas will vary depending on national context and priorities. Some countries may focus on disease burden and implementation/operational research to inform new product implementation, whereas others may have wider-ranging agendas. |
| Method of measurement | Proposed JRF Questions:   - Do you have a national agenda for research on immunization?   **IF YES,**  - Please provide the supporting document (e.g. national immunization strategy, national health plan) which can provide an evidence of national agenda for research on immunization  From this self reported and supporting documentation, a desk review will be conducted to:   - Establish the baseline of how many national immunization research agendas currently exist, what form are they in (how variable), where are they situated, how are they monitored (desk review, surveys through ROs?) - Assess whether a framework or guidance for developing national immunization strategies is desirable, useful – and develop one if needed - Assess progress towards national immunization strategy as part of NIS reporting |
| Data source | Primary data source   - Proposed to be collected through JRF   Countries should review these sources for the document of their research agenda   - National Immunization Technical Advisory Groups - National Immunization strategies - National regulatory bodies - RITAGs - Clinical trial registeries |

| **Indicator ID, Name** | **SP 7.2- Progress towards global research and development targets** |
| --- | --- |
| Definition | Progress towards global research and development targets will be monitored based on “short list” of global targets which will be developed by WHO and endorsed by SAGE |
| Calculation and operational considerations | WHO HQ and regional offices together with key partners/stakeholders will mutually define targets and monitor and evaluate progress at the global and regional level. The process will require a prioritization framework to align on priorities, targets, and a mechanism for monitoring and evaluation.  The suggested short list should be presented no later than SAGE Oct 2022. |
| Method of measurement | **Global**: Measurement will require   - Periodic review of literature to track topical trends and progress - Baseline will be established through the same process using periodic review of literature |
| Data source | Primary data source:   - Periodic review of literature, including grey literature   Review of literature should include the following sources:   - WHO Product Development and Vaccine Advisory Committee and associated working groups (https://www.who.int/immunization/research/committees/pdvac/en/) - Vaccine Innovation Prioritization Strategy (https://www.gavi.org/our-alliance/market-shaping/vaccine-innovation-prioritization-strategy) - Infuse (<https://www.gavi.org/investing-gavi/infuse>) - Clinical trial registries, manufacturers websites, product pipelines etc, ethics research review |

# Further Development of Impact Goal and Strategic Priority Objective Indicators

The IA2030 M&E Framework includes several impact goal (IG) and strategic priority (SP) objective indicators that were not previously collected and need further development. In addition, the Learning Agenda indicates that the M&E Framework should be reviewed and updated at least once every three years in response to changing programmatic needs and improvements in M&E methods, to ensure it delivers the data required to improve immunization programme performance. This periodic review should assess if the collected data are fit for purpose and make necessary revisions to update the indicators as immunization programme capacities are continuously strengthened.

The following impact goal indicators need further development, including additional data collection and/or in-depth analyses of historical trends and projections (e.g. the anticipated long-term impact of the COVID-19 pandemic) to produce baseline estimates and to set realistic targets.

**IG 1.1** Number of deaths from vaccine-preventable diseases averted.

- Over the next few years, additional pathogens (p.22) will be added to the scope of IG 1.1. These pathogens were categorized based on strategic priorities, data availability and feasibility.
- Estimates for IG 1.1. will be updated and reported on an annual basis with the WUENIC release.
- The models and the methodology will be further refined, and estimates will be validated with additional data.
- The anticipated impact of the COVID-19 pandemic on coverage rate will be incorporated as more data become available.

**IG 1.2** Number and proportion of countries that have achieved global or regional VPD control, elimination and eradication targets.

- Additional VPDs may be included for analysis as regional or global bodies endorse new VPDs for control, elimination and eradication.
- Updates to both the VPD indicators and the VPD targets will be made as disease programmes’ monitoring strategies evolve. Possible updates include update from regional endorsement to global endorsement, target value, target type and target date timeframe.

**IG 1.3** Number of large or disruptive outbreaks of vaccine-preventable diseases.

- Criteria for large or disruptive outbreaks of measles, polio, meningococcus, yellow fever, cholera, and Ebola will be developed in alignment with global vaccine-preventable disease strategies. These criteria will be updated as needed during the decade to reflect changes in vaccine-preventable disease strategies.
- Historical and baseline disease surveillance data will be assessed against the finalized criteria. Assessments of the number of outbreaks qualifying as large or disruptive outbreaks will be updated annually for each disease within scope of IG 1.3.
- Over the next few years, additional pathogens may be added to the scope of IG 1.3 in alignment with global vaccine-preventable disease eradication, elimination and control goals, particularly as additional outbreak-prone diseases become vaccine-preventable.

**IG 3.2** UHC Index of Service Coverage (UHC SCI)

- The metrics used to measure UHC and PHC will likely evolve over the decade with advances in data and understanding. The target for the IG3.2 indicator will also evolve to ensure immunization programme’s contribution to PHC/UHC is best measured.

In addition, all of the IG indicators need to be assessed as they are implemented to properly collect, measure, analyse, interpret, communicate and use the results to drive progress to achieve the IA2030 impact goals.

The following SP objective indicators need further development:

**SP Objective 1.1**: Reinforce and sustain strong leadership, management and coordination of immunization programmes at all levels.

- Well-functioning monitoring, evaluation and action cycles to continuously improve immunization programme quality are a key proxy measure of leadership, management and coordination. Monitoring of this indicator might require development of new reporting and feedback mechanisms and capacity building for implementation of ME&A cycles at all levels.

**SP Objective 2.2**: Ensure that all people and communities value, actively support and seek out immunization services.

- This indicator is intended to drive national immunization programmes to allocate dedicated resources to assess and address barriers to vaccination. However, it was not feasible to develop a single global demand creation indicator that is applicable to all countries, and the availability of data to measure this indicator might be a challenge in some countries.

**SP Objective 3.1:** Ensure preparation for, detection of, and rapid, high-quality responses to vaccine-preventable disease outbreaks.

- Criteria for timely outbreak detection and response will be finalized for each disease in scope of SP5.1 in alignment with global vaccine-preventable disease eradication, elimination and control goals.
- Historical and baseline disease surveillance data will be assessed against the finalized criteria. Assessments of the proportion of outbreaks with outbreak response vaccination campaigns that had timely outbreak detection and response will be updated annually for each disease within scope of SP 5.1. Criteria for timely outbreak detection and response may be revised periodically in light of new diagnostic technology and methodologies for disease surveillance and outbreak response.
- Over the next few years, additional pathogens may be added to the scope of SP5.1 in alignment with global vaccine-preventable disease eradication, elimination and control goals, particularly as additional outbreak-prone diseases become vaccine-preventable.

**SP Objective 7.2**: Develop new vaccines and associated technologies, and improve existing products and services for immunization programmes

- This indicator from GVAP (i.e. a short list of global priority R&D targets) is intended to be an interim indicator until a strategic approach to set R&D agendas for development of new vaccines and technologies, and improvements of existing products and services for immunization programmes, is defined through global and regional mechanisms.
- The strategic approach to set R&D agendas should consider national agendas for immunization research (SP7.1), and reflect the IA2030 Research & Innovation strategy which focuses on “needs-based innovation and aims to strengthen mechanisms to identify research and innovation priorities according to community needs, particularly for the under-served, and ensure these priorities inform innovations in immunization products, services and practices.” Regional R&D agendas should be focused on achieving the greatest impact among countries in the region; regional R&D agendas should feed into the global R&D agenda, ensuring that the global R&D agenda is anchored in the needs of communities.

1. 1. Vaccine antigens included: HepB, Hib, HPV, JE, measles, MenA, Streptococcus pneumoniae, rotavirus, rubella, yellow fever, diphtheria, tetanus, pertussis, BCG. Measured relative to zero coverage levels (absence of vaccination); target includes deaths averted over the lifetime of the birth cohort by vaccines given during 2021-30.

   2. Eradication (polio), elimination of transmission (measles, rubella), elimination as a public health problem (HPV, MNT, hepatitis B), control (Japanese encephalitis)

   3. Large or disruptive outbreaks of measles, polio, meningococcus, yellow fever, cholera, and Ebola will be defined based on criteria for each disease.

   4. Vaccines included: HepB birth dose, Hib, HPV, IPV2, MCV2, PCV, rotavirus, rubella, DTP booster, COVID-19, JE, YF, MenA, multivalent meningitis, typhoid, cholera, dengue, rabies, HepA, influenza, varicella, and mumps. Malaria and other relevant vaccines will potentially be included when recommended.

   5. COVID-19 vaccination coverage will potentially be included. [↑](#footnote-ref-1)
2. Guidance for selection of regional and country strategic priority objective indicators is provided below under the section “Strategic Priority Objective Indicators. [↑](#footnote-ref-2)
3. Disease-specific initiatives include: GPEI Polio Endgame Strategy 2021–2026; Measles and Rubella Strategic Framework 2021–2030; Ending Cholera – A Global Roadmap to 2030; Global Health Sector Strategy on Viral Hepatitis 2016–2021; Defeating Meningitis by 2030 Roadmap; Global Influenza Strategy 2019–2030; Zero deaths from dog-mediated rabies by 2030 (Zero by 30: The Global Strategic Plan); Achieving and sustaining maternal and neonatal tetanus elimination: Strategic Plan 2012-2015; Global Vector Control Response 2017–2030; Eliminate Yellow Fever Epidemics 2017-2026. Those included in the indicator may evolve over time with new regional and/or global endorsement. [↑](#footnote-ref-3)
4. Disease-specific initiatives include: GPEI Polio Endgame Strategy 2021–2026; Measles and Rubella Strategic Framework 2021–2030; Ending Cholera – A Global Roadmap to 2030; Defeating Meningitis by 2030 Roadmap; Eliminate Yellow Fever Epidemics 2017-2026. [↑](#footnote-ref-4)
